# Dysregulation of systemic immunity and its clinical application in gastric cancer

**DOI:** 10.3389/fimmu.2024.1450128

**Published:** 2024-09-05

**Authors:** Yao Zhang, Junfeng Li, Jian Li, Jisheng Wang

**Affiliations:** ^1^ Department of General Surgery, The Third Hospital of Mianyang, Sichuan Mental Health Center, Mianyang, Sichuan, China; ^2^ School of Pharmacy, Southwest Medical University, Luzhou, Sichuan, China; ^3^ Department of Pharmacy, The Third Hospital of Mianyang, Sichuan Mental Health Center, Mianyang, Sichuan, China

**Keywords:** gastric cancer, systemic immunity, detection, prognosis, cancer therapy

## Abstract

Immunotherapy has profoundly changed the treatment of gastric cancer, but only a minority of patients benefit from immunotherapy. Therefore, numerous studies have been devoted to clarifying the mechanisms underlying resistance to immunotherapy or developing biomarkers for patient stratification. However, previous studies have focused mainly on the tumor microenvironment. Systemic immune perturbations have long been observed in patients with gastric cancer, and the involvement of the peripheral immune system in effective anticancer responses has attracted much attention in recent years. Therefore, understanding the distinct types of systemic immune organization in gastric cancer will aid personalized treatment designed to pair with traditional therapies to alleviate their detrimental effects on systemic immunity or to directly activate the anticancer response of systemic immunity. Herein, this review aims to comprehensively summarize systemic immunity in gastric cancer, including perturbations in systemic immunity induced by cancer and traditional therapies, and the potential clinical applications of systemic immunity in the detection, prediction, prognosis and therapy of gastric cancer.

## 1 Introduction

Although its incidence has decreased in recent decades, gastric cancer (GC) remains the most common cause of cancer-related death worldwide, especially in regions with high *Helicobacter pylori* infection, such as East Asia, South America and the Middle East (1). In 2022, both the number of new cases of GC worldwide and the number of GC-related deaths ranked fifth, with estimated values of 968350 and 659853, respectively (2). Except in some countries and regions with well-established screening programs, such as Japan, Korea and some areas in China, the majority of GC patients are diagnosed at late stages, leading to dismal long-term survival (3).

Traditionally, the main strategy for curing GC is radical gastrectomy, and in some cases, chemotherapy and/or radiotherapy are needed to reduce recurrence. However, in unresectable advanced patients, systemic chemotherapy combined with targeted therapy is the standard treatment, aiming to prolong survival and improve quality of life (3). The participation of the immune system in tumorigenesis has long been heavily investigated, resulting in the impressive success of immunotherapy in the past decade. Immunotherapy, including immune checkpoint inhibitors (ICIs), cell-based therapy and vaccines, has revolutionized cancer therapy, and several recently published phase 3 clinical trials have proven the encouraging effects of immunotherapy in patients with GC in first- or late-line settings (4–7). Nevertheless, patients who achieve a durable response are limited, while the majority of patients with GC are primarily or secondarily resistant to immunotherapy. Therefore, numerous efforts have been devoted to elucidating the mechanisms underlying the responsiveness of GC to immunotherapy. However, most of these studies are limited to the tumor microenvironment (TME), such as programmed cell death-ligand 1 (PD-L1) expression, the tumor neoantigen load and the profile of infiltrating immune cells (8, 9).

Although perturbations in systemic immunity have long been observed in cancer patients, how they influence the progression of tumors and the effects of cancer therapies, especially immunotherapy, have not received much attention until recently (10). Inspired by these findings, this review aimed to focus on systemic immunity in GC. We first summarize the perturbations of systemic immunity induced by GCs and then outline the effects of traditional therapies, including radical gastrectomy and chemotherapy, on systemic immunity. Finally, we address the potential clinical applications of systemic immunity in the detection, prediction, prognosis and therapy of GC.

## 2 Perturbations in systemic immunity induced by GC

**Figure 1:**
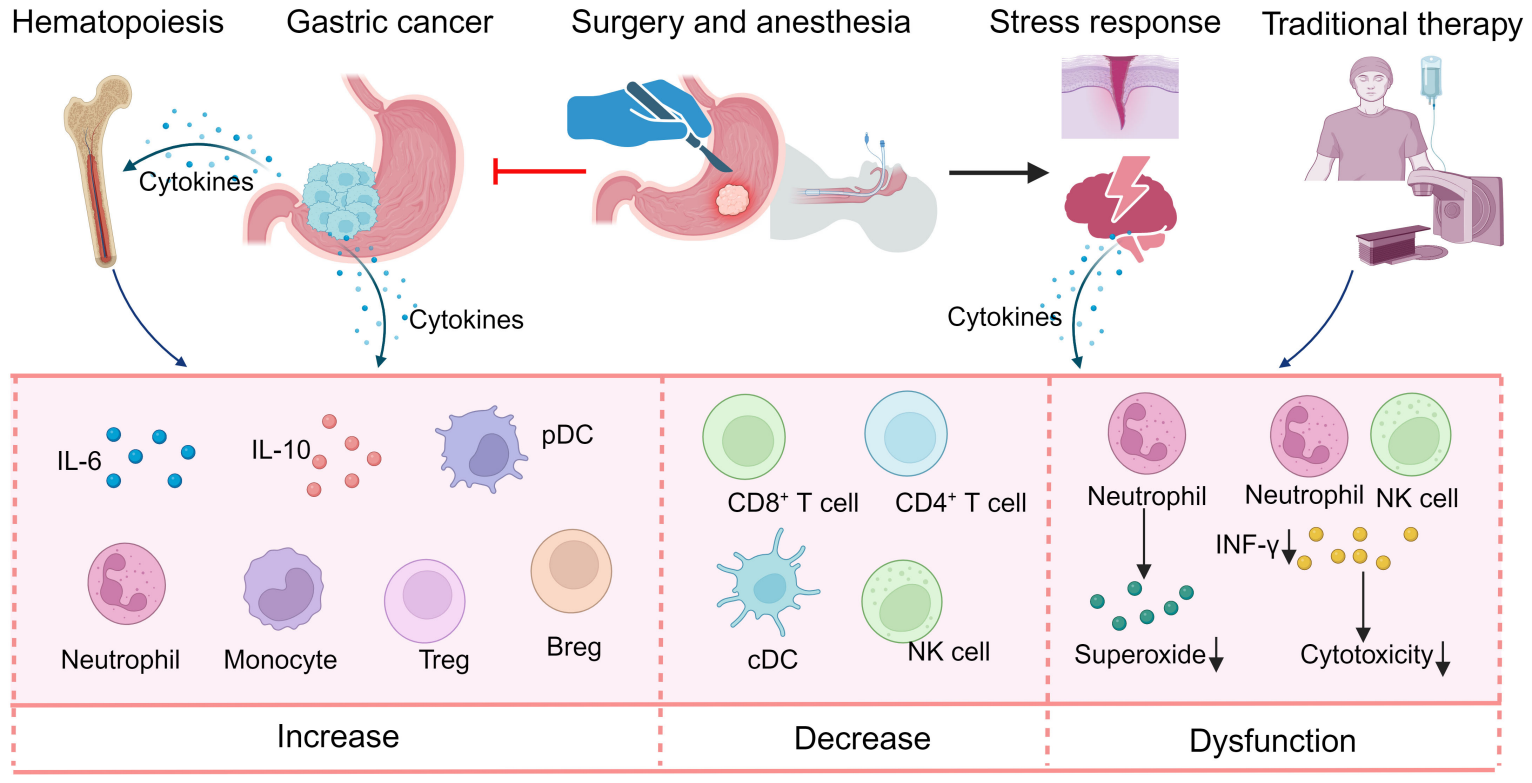
Perturbations in systemic immunity in gastric cancer. Gastric cancer itself and traditional therapy can induce reorganization of systemic immunity, manifesting most prominently in alterations in circulating cytokines, expansion of immunosuppressive myeloid populations and a decrease in immune cells with antitumor ability. Breg, regular B cell; cDC, classical dendritic cell; INF, interferon; NK, natural killer; pDC, plasmacytoid dendritic cell; Treg, regular T cell.

Through the disruption of hematopoiesis or direct effects on peripheral immune cells, both human cancers and animal tumor models have been shown to induce extensive perturbations in systemic immunity, manifesting as alterations in circulating cytokines, the expansion of immunosuppressive myeloid populations and a decrease in immune cells with antitumor ability (11, 12). Progenitors with myeloid differentiation potential have been found to increase in the bone marrow of mouse models, leading to elevated frequencies of neutrophils and monocytes, along with reductions in dendritic cell (DC) and lymphocyte populations, which can be reversed by resection of cancer or cytokine blockage, suggesting that circulating cytokines secreted by cancer cells drive the remodeling of systemic immunity (12). Currently, no studies have investigated the changes in hematopoiesis in bone marrow induced by GCs; however, numerous studies have reported perturbations in cytokines and major immune lineages in peripheral blood (**Figure 1**; **Supplementary Table S1**).

### 2.1 Circulating cytokines

Cytokines are a collection of molecules that participate in almost every step of tumorigenesis and immunology (13). Numerous studies have investigated the perturbations of circulating cytokines in GC patients (14). In general, the concentrations of cytokines, including interleukin-1β (IL-1β), IL-6, IL-10, IL-17, interferon-γ (IFN-γ) and tumor necrosis factor-α (TNF-α), in the peripheral blood of GC patients are commonly greater than those in healthy subjects, whereas the concentration of macrophage chemotactic protein (MCP)-1 has been found to be lower in several studies (15–19). However, cytokines in the circulation can be secreted by nearly all cells, including immune cells and GC cells themselves, and their levels are strongly affected by various factors, such as age, sex, lifestyle and genetic background (20–23). Therefore, inconsistent findings are common across studies, and accurately interpreting the perturbations in circulating cytokines induced by GCs is difficult. For individual cytokines, the peripheral changes in GC patients may be context dependent rather than a regular phenomenon. For example, although IL-6 is one of the most common cytokines whose peripheral levels are increased in GC patients, several studies have not shown significant differences between GC patients and controls (16, 24–26). Furthermore, the serum levels of several cytokines are associated with clinicopathological features. For example, IL-6 levels are elevated only in intestinal GC patients, whereas MCP-1 levels are lower only in diffuse GC patients (16). Nevertheless, a general finding is that the degree of alterations in peripheral cytokines increases with disease progression (21, 27, 28).

### 2.2 Myeloid lineages

Several cancers have been demonstrated to promote hematopoiesis toward monocytic and granulocytic lineages through cancer-derived factors (10). Although such hematopoietic alterations have not been validated in GC, many studies have reported that both neutrophils and monocytes are extensively perturbed in GC patients. However, these perturbations are commonly represented by ratios between different immune cells, while studies on their functions are limited. In general, patients with GC have a greater percentage of neutrophils in the peripheral blood than healthy donors do, and with increasing tumor burden, the percentage of neutrophils in the periphery significantly increases (29, 30). Although disparities in phenotype and function have been found between neutrophils obtained from cancer tissues and peripheral blood, these differences between neutrophils from GC patients and those from healthy subjects have not been addressed (29). In terms of function, peripheral neutrophils from GC patients exhibit normal phagocytic activity but reduced superoxide generation (31). Furthermore, a subset of myeloid-derived suppressor cells (MDSCs), which highly express neutrophil markers, is dramatically increased in the circulation of GC patients and has the ability to suppress the activity of CD8+ T cells (32). In addition, neutrophil extracellular traps (NETs), one of the main contributors to the cancer-promoting ability of neutrophils, were found to be more abundant in the blood of GC patients, especially those with late-stage disease (33, 34). Another cell type of myeloid origin, monocytes, was also found to be increased in the peripheral blood of GC patients with decreased chemotactic responsiveness and upregulated T-cell immunoglobulin and mucin-domain containing-3 (TIM-3) expression, which may be an important mechanism in GC progression (35, 36). In addition to these findings, few studies have investigated GC-induced dysfunction of neutrophils and monocytes. As both neutrophils and monocytes are composed of heterogeneous cell populations with pro- and anticancer abilities, determining their alterations is important for clinical applications.

Dendritic cells, which participate in antigen presentation and T-cell priming and proliferation, are critical orchestrators of innate and adaptive immunity in cancer (37). Therefore, perturbation of DCs from the peripheral circulation has long been observed in many cancers, including GC (10). Compared with healthy individuals, patients with GC have lower circulating DC counts and percentages, with reduced cytotoxicity and TNF-α, IL-2 and CD40 expression, indicating impaired function and immature status (38, 39). However, similar to other immune cells, DCs are composed of various heterogeneous subsets, including plasmacytoid DCs (pDCs), classical DCs (cDCs) and inflammatory DCs, which play different roles in human diseases (40). Therefore, despite the overall decrease in peripheral DCs, subsets with tumor-promoting effects, such as pDCs and DC-10 cells, were found to be elevated in the periphery of GC patients (41–43). In patients with GC, the frequency and mean fluorescence intensity of DC-10 in the peripheral blood are dramatically increased and strongly associated with tumor grade (41). The levels of other tolerogenic DCs, pDCs, are also significantly increased in the blood of GC patients, and these pDCs are proposed to be recruited to the TME through chemokine receptor 9 (CCR9) and C-C motif chemokine (CCL25) interactions, leading to an immunosuppressive microenvironment in GC (42). Therefore, subsets and functional states should be taken into consideration when peripheral DCs are utilized for clinical application.

### 2.3 Lymphoid lineage

As the main participants in antitumor immunity, lymphocytes have long been the focus of cancer immunology. In terms of peripheral immune cells in GC, substantially more studies have investigated alterations in lymphocytes. Despite a few inconsistent findings, lymphopenia is common in GC patients (15, 44). However, circulating lymphocytes are composed of complicated subsets with both cancer-promoting and cancer-inhibiting activities. Therefore, in addition to the total lymphocyte population, individual subsets have also been extensively studied. CD8+ T cells are the main effector cells involved in tumor cell killing, and their functions are strongly impaired in the peripheral blood of GC patients (45, 46). Under some conditions, CD8+ T cells can be induced by GCs to express IL-10, PD-1 and TIM-3, which inhibit the effector function of CD8+ T cells (46, 47). In contrast, the levels of suppressive lymphocytes, such as regulatory T (Treg) cells, regulatory B (Breg) cells and IL-17-producing T cells, are typically greater in GC patients than in normal controls (48–52). These cell types synergize with each other to establish an immunosuppressive environment in GC patients. For example, increased Breg cells in the blood of GC patients inhibited the production of T-cell cytokines and converted T cells to Treg cells, leading to immune escape in GC (52).

Natural killer (NK) cells are a type of innate immune cell that differentiates from common lymphoid progenitors and participates in cancer immunosurveillance through direct cancer cell killing and orchestrates the functions of other players in the immune system (53). The phenotype of circulating NK cells from GC patients differed from that from healthy controls, characterized by a decrease in the number of NK cells expressing activating molecules, including NKp30, NKp46, NKG2D and DNAM-1, and an increase in the number of NK cells expressing the inhibitory molecules KIR3DL1 and TIM-3; these perturbations are significantly associated with cancer progression (54–56). Additionally, the anticancer capacity of these NK cells has been shown to be impaired in GC patients, manifesting as decreased IFN-γ production and cytotoxic function (21, 57, 58).

### 2.4 Indices derived from multiple immune components

The human immune system is an intricate network with complex synergistic and/or antagonistic interactions among individual immune components. Therefore, various indices derived from multiple immune components have been established to reflect the peripheral immune state more precisely. Owing to their low cost and noninvasive accessibility, numerous studies have investigated alterations in these indices in patients with GC. In general, compared with healthy individuals, patients with cancer present with a greater neutrophil-to-lymphocyte ratio (NLR), platelet-to-lymphocyte ratio (PLR), and systemic immune-inflammation index (SII), and lower lymphocyte-to-monocyte ratio (LMR), and the severity of perturbations increases with disease progression (15, 59–61).

Overall, these findings strongly indicate that systemic immunity, which results from the effects of cancer cells and participates in the progression of cancer, is reduced in GC patients. Further efforts are warranted to fully clarify the systemic immune landscape of patients with GC and its associations with disease stage and patient demographics. The mechanisms underlying these perturbations are also largely unknown in GC, and elucidating these mechanisms is critical for therapeutic development.

## 3 Changes in systemic immunity during traditional therapy for GC

Traditional therapies for GC, including surgery, chemotherapy, and radiotherapy, have long been known to positively or negatively affect systemic immunity, which may determine the efficacy of treatment and oncological outcomes (**Supplementary Table S2**). In contrast, although targeted therapy has been developed as a standardized treatment for GC in various settings and research on its combination with immunotherapy has been increasing, few studies have been conducted to investigate the effects of targeted therapy on systemic immunity in patients with GC. Elucidating the changes in systemic immunity during traditional GC therapy is critical for optimizing these strategies to strengthen rather than impair anticancer immunity.

### 3.1 Radical gastrectomy and perioperative events

Radical gastrectomy is the main method for achieving complete disease control and long-term disease-free survival in GC patients. As mentioned above, systemic immune perturbations are induced mainly by various factors derived from the primary tumor; therefore, radical gastrectomy can eliminate these factors and restore normal peripheral immunity, which has been demonstrated in breast and colon cancer (12). However, wound healing and the stress response following gastrectomy also have detrimental effects on systemic immunity.

After radical gastrectomy, wound healing programs remodel systemic immunity, characterized by elevated circulating IL-2, IL-6, IL-10, TNF-α and IFN-γ, ultimately driving peripheral immune cells to immunosuppressive states (62–64). In addition to gastrectomy, perioperative events, including anesthesia, analgesia, postoperative complications, intraoperative blood loss and blood transfusion, all activate or prolong the surgical stress response, leading to the activation of neural signaling and systemic inflammation (65). More extended surgery and an eventful postoperative course are associated with elevated serum catecholamines, which have been shown to suppress anticancer immunity (66). Therefore, the peripheral IL-6 concentration is greater in GC patients who have undergone longer operations (62). In patients who underwent gastrectomy in combination with splenectomy, the T-cell subsets were decreased, and their functions were significantly suppressed (64). The number and cancer cell-killing potential of NK cells are also decreased by abdominal laparotomy, which leads to lung metastasis (67). In addition to the direct suppression of anticancer effector immune cells, surgical stress also increases the levels of immune inhibitory molecules and cells in the periphery. For example, in mouse models, gastrectomy can induce the accumulation of γδT cells in mesenteric lymph nodes, which suppresses the cell-mediated response by transforming growth factor-β (TGF-β) (68). Although some studies have shown that increased proinflammatory cytokines return to normal levels immediately after gastrectomy, long-term functional suppression of immune cells in the blood has been demonstrated in breast cancer models (62, 63, 69). Collectively, these findings suggest that gastrectomy and perioperative events can induce systemic immune perturbations.

### 3.2 Cytotoxic therapy

The majority of chemotherapeutics and radiation kill cancer cells through direct damage and induction of apoptosis. Although the latter mechanism may cause immunogenic death, which enhances anticancer immunity, acute cancer cell death and the stress response of cells within the TME release various proinflammatory molecules into the circulation to modulate the function of peripheral immune cells (70). Currently, although chemotherapy and radiotherapy are known to cause granulopenia and lymphopenia in clinical practice, studies investigating the effects of cytotoxic therapy on systemic immunity in GC patients are scarce. Few studies have suggested that following chemotherapy, the levels of immune-enhancing cytokines, including IL-2, IL-4, IL-10, and IFN-γ, decrease in the serum of GC patients (15, 71). Nevertheless, the most commonly used cytotoxic drugs in GC, such as oxaliplatin and 5-fluorouracil (5-FU), were found to reorganize systemic immunity in patients with cancer. For example, oxaliplatin induces the systemic release of ectoenzyme-expressing extracellular vesicles (EVs) from B cells and ATP from cancer cells, leading to the production of adenosine, which contributes to CD8+ T cell dysfunction (72). In addition, in response to 5-FU, circulating proinflammatory factors secreted by myeloid and CD4+ T cells promote tumor progression (73, 74). Whether these impairments also occur in patients with GC needs further clinical study.

## 4 Potential for clinical application

### 4.1 Roles of systemic immunity in cancer progression

**Figure 2:**
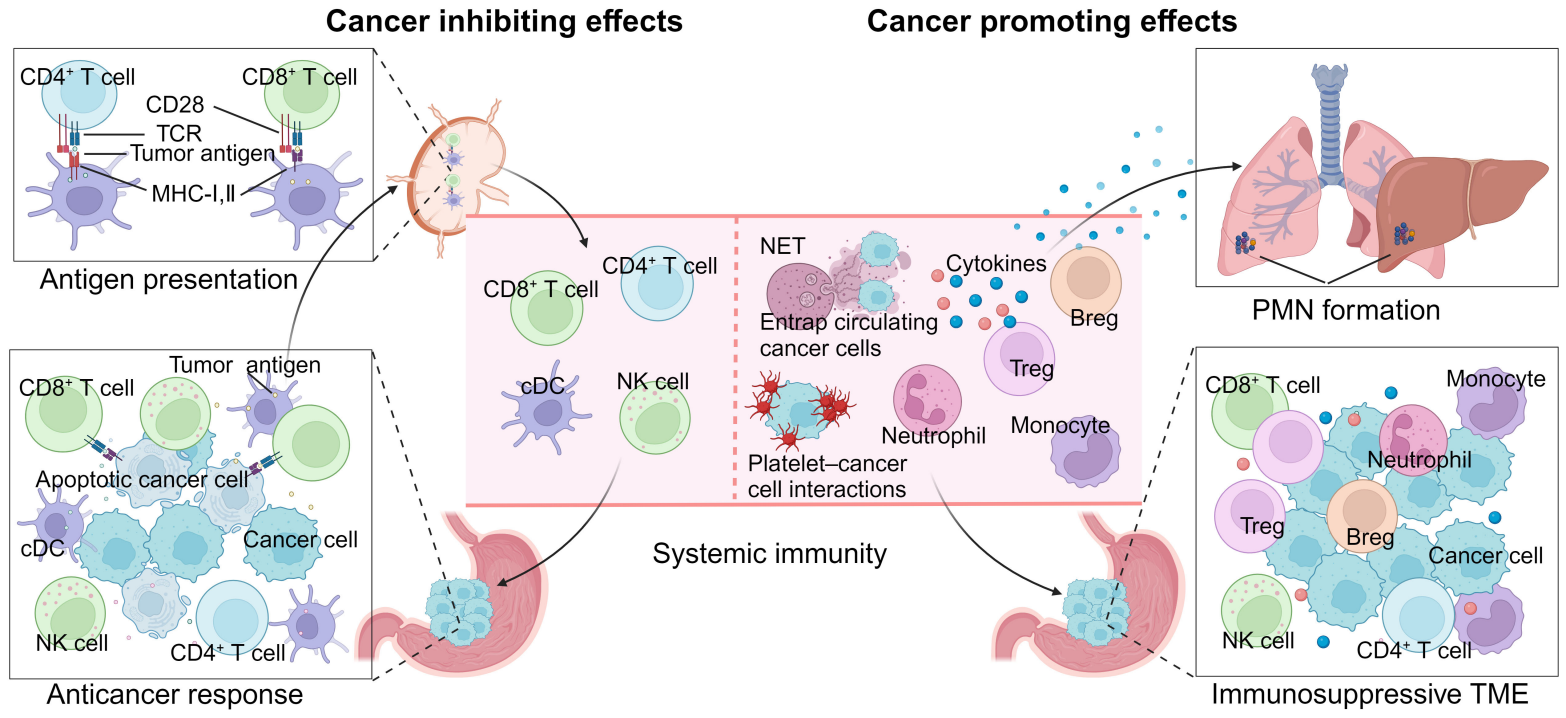
Roles of systemic immunity in gastric cancer progression. On the one hand, intact peripheral immunity is essential for the anticancer immune response, as the majority of steps of the tumor-immune cycle occur outside the tumor microenvironment (TME). On the other hand, dysregulated systemic immunity promotes cancer progression through immunosuppressive TME development, premetastatic niche (PMN) formation and circulating tumor cell protection. Breg, regular B cell; cDC, classical dendritic cell; MHC, major histocompatibility complex; NET, neutrophil extracellular trap; NK, natural killer; PMN, premetastatic niche; TME, tumor microenvironment; TCR, T-cell receptor; Treg, regular T cell.

Multiple decades of research have demonstrated that the immune system has both cancer-promoting and cancer-inhibiting functions, the process of which is referred to as cancer immunoediting and comprises three phases: elimination, equilibrium and escape (75). However, the conceptual development has been based mainly on the TME, whereas the role of systemic immunity has been less considered. For successful cancer cell elimination by natural and therapeutically induced anticancer immunity, intact peripheral immunity is a critical determinant, as the majority of steps of the tumor-immune cycle, including tumor antigen presentation, effector cell priming, proliferation and trafficking, occur outside the TME (76). Therefore, when the progression of effector cells is blocked or cancer drainage lymph nodes are resected, immunotherapeutic efficacy is abrogated (77, 78). On the other hand, through various secreted factors, many cancers can disrupt hematopoiesis extensively and drive circulating immune cells toward accomplices to facilitate tumor progression (10). In addition to preresident cells, the majority of cancer-infiltrated immune components circulate from the periphery and participate in the development of an immunosuppressive TME. For example, a strong association was observed between the levels of peripheral and intratumoral neutrophils, indicating that the expanded immature neutrophils in the peripheral blood of GC patients also infiltrate cancer tissues, resulting from the high expression of molecules involved in neutrophil recruitment and plasticity modulation (79). Furthermore, perturbed systemic immunity is involved in many steps of the cancer invasion-metastasis cascade. For example, cancer-edited immune cells induce the formation of premetastatic niches that are conducive to the survival and proliferation of cancers before their arrival (80). During trafficking in the circulation, cancer cells are protected and supported by many immune components, including neutrophils and platelets (81, 82) (**Figure 2**).

**Figure 3:**
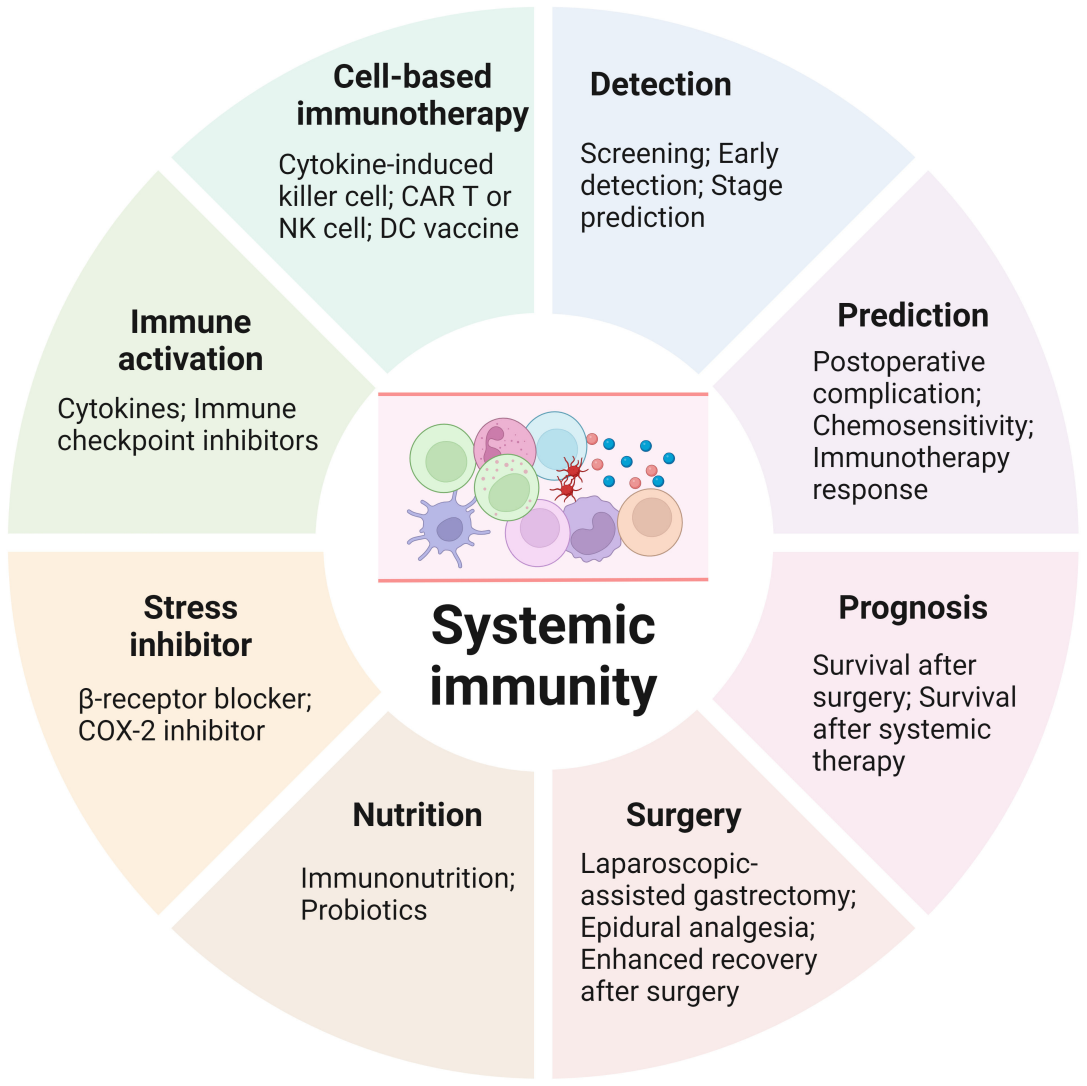
Clinical applications of systemic immunity in gastric cancer. Biomarkers based on systemic immunity can be utilized for gastric cancer detection, therapy response prediction and prognosis. Various strategies have been shown to preserve or activate systemic immunity in patients with gastric cancer. Furthermore, cells from systemic immunity are the main sources of cell-based immunotherapies. CAR, chimeric antigen receptor; COX-2, cyclooxygenase; DC, dendritic cell; NK, natural killer.

Because of its critical roles and extensive perturbations that occur during cancer progression, the potential of systemic immunity for GC detection, efficacy prediction, prognosis and therapy has been extensively investigated (**Figure 3**).

### 4.2 Detection

As mentioned above, compared with healthy subjects, GC patients exhibit extensive alterations in peripheral immune components. Owing to their low cost and noninvasive accessibility, the potential of these alterations as biomarkers for GC detection has been explored. For example, the serum MIC-1 is significantly elevated in early GC, and the performance of early GC detection was 72.9% (83). A diagnostic model including CEA, CA724, IL-6, IL-8, and TNF-α showed the potential to screen for GC, including patients with early-stage disease (84). Peripheral immune cells and indices derived from them also have diagnostic value for GC (41, 59, 85). In addition, the preoperative NLR is significantly correlated with the presence of peritoneal metastasis, especially for type 4 or diffuse type 3 cancers, which may have potential in decision-making regarding staging laparoscopy (86). Despite these promising findings, the application of these biomarkers for the screening and identification of high-risk populations for GC is still lacking. First, all the data were obtained from retrospective cohorts, the majority of which had small sample sizes and were not validated prospectively or externally. Second, the cut-offs used to define high or low levels varied across studies, impeding the development of an optimal method for generalization. Third, peripheral immune components are widely affected by various factors, leading to low specificity for the detection of GC. Therefore, systemic immunity-related biomarkers may be utilized as supplements rather than methods to screen for GC independently.

### 4.3 Efficacy prediction

Treatment for GC, including surgery, chemotherapy, radiotherapy, targeted therapy and immunotherapy, puts patients at risk for complications and adverse effects. Therefore, exactly predicting the efficacy and possible adverse effects has long been a goal in the management of GC. Postoperative complications following radical gastrectomy, especially infections, including anastomotic leakage, pneumonia and intraabdominal infections, are significantly associated with oncological outcomes (65, 87). Therefore, various prediction models, including indices based on components of systemic immunity, have been established to assess the risk of postoperative complications. For example, preoperative peripheral T cells, B cells, NK cells, the NLR, the PLR and the LMR are predictive of prolonged hospital stays and infectious complications (88, 89). Furthermore, the postoperative systemic immune state is also a predictor of infectious complications (90). Nevertheless, whether preoperative strategies based on these prediction models will improve the short-term outcomes of GC patients has not been investigated. In terms of chemotherapy and immunotherapy alone or in combination, which are used to reduce recurrence following radical gastrectomy or to prolong the survival of patients with advanced disease, the predictive value of biomarkers based on peripheral immune components has also been suggested. The PLR obtained prior to chemotherapy might be a useful indicator for predicting chemosensitivity, whereas the baseline IL-6 concentration and progressive decrease in the PLR during treatment can predict the therapeutic benefits of immunochemotherapy (91–94). Furthermore, baseline circulating Treg cell levels can predict the probability of the occurrence of immunotherapy-related adverse events (95). In the future, if these predictive biomarkers can be regularly and dynamically monitored, personalized therapy for GC can optimize therapeutic effects while reducing adverse events.

### 4.4 Prognosis

A precise prognosis is critical in the management of GC patients, as more intensive therapy may be needed for patients with negative prognostic factors. Therefore, numerous host and cancer features, including biomarkers based on systemic immunity, have been explored as prognostic factors. In general, higher levels of peripheral immune components involved in anticancer responses indicate better survival. For example, IL-2 and INF-γ levels are positively associated with overall survival (OS), whereas IL-6, IL-10 and IL-17A levels are negatively associated with OS (15, 24, 96). C-reactive protein (CRP), an indicator of ongoing proinflammatory response, was negatively associated with OS in a meta-analysis (97). In addition, ICOS+Foxp3+ Treg cells and pDCs in the peripheral blood could predict poor clinical outcomes in GC patients (98). In the literature, the most studied systemic immune biomarkers with prognostic value in GC are indices derived from multiple immune components, such as the NLR, PLR, LMR and prognostic nutritional index (PNI). **Table 1** summarizes the results of the meta-analyses on the prognostic value of these indices in patients with GC receiving different treatment strategies. These meta-analyses consistently suggest that higher levels of neutrophils, monocytes and platelets and lower levels of lymphocytes are significantly associated with poor OS (**Table 2**) (91, 97, 99–122).

**Table 1:** Meta-analyses investigating the prognostic value of systemic immune biomarkers in gastric cancer.

Author	Year	Treatment	Index	Cutoff	OS			Reference
					No. of comparison	HR (95% CI)	I^2^	
Szor DJ	2018	Surgery	NLR	1.40-4.02	7	2.89 (2.41-3.47)	85%	(99)
Mellor KL	2018	Surgery	NLR	1.44-5.5	5	2.31 (1.40-3.83)	84%	(100)
Li LL	2023	Immunotherapy	NLR	2.5-5.0	10	2.13 (1.70-2.66)	13%	(101)
Zhang S	2023	Immunotherapy	NLR	2.5-5.0	9	1.98 (1.67-2.35)	19%	(102)
Matsas S	2024	Immunotherapy	NLR	2.5-5.0	10	2.11 (1.70-2.62)	45%	(103)
Du S	2021	Systemic therapy	NLR	2.5-5.0	36	1.78 (1.59-1.99)	80%	(104)
Sun J	2016	Not specific	NLR	1.44-5	19	1.98 (1.75-2.24)	53%	(105)
Kim MR	2020	Not specific	NLR	1.44-5.0	24	1.61 (1.45-1.78)	51%	(91)
Xu Z	2016	Surgery	PLR	126-184	7	0.99 (0.89-1.10)	12%	(106)
Matsas S	2024	Immunotherapy	PLR	139.41-267.00	5	1.77 (1.44-2.17)	25%	(103)
Chen J	2015	Chemotherapy	PLR	2.15-5.0	9	2.16 (1.86-2.51)	65%	(107)
Hu G	2022	Chemotherapy	PLR	107.7-284	11	1.60 (1.41-1.82)	39%	(108)
Peng X	2022	Chemotherapy	PLR	107.7-284	16	1.43 (1.25-1.64)	54%	(97)
Zhang X	2014	Not specific	PLR	NR	10	1.83 (1.62-2.07)	30%	(109)
Zhang X	2020	Not specific	PLR	10.1-350	44	1.37 (1.26-1.49)	80%	(110)
Cao W	2020	Not specific	PLR	108-350	28	1.37 (1.24-1.51)	68%	(111)
Gu X	2016	Not specific	PLR	126-235	14	1.30 (1.10-1.52)	69%	(112)
Ma JY	2018	Surgery	LMR	3.15-5.15	6	0.66 (0.54-0.82	75%	(113)
Mei P	2023	Immunotherapy	LMR	2.8-5.0	7	0.51 (0.33-0.79)	55%	(114)
Yang Y	2016	Surgery	PNI	45-49.7	10	1.89 (1.67-2.13)	7.40%	(115)
Li J	2019	Surgery	PNI	40-52	15	1.81 (1.56-2.09	49%	(116)
Yang X	2024	Not specific	SII	315-1185.2	27	1.53 (1.34-1.75)	72.40%	(117)
Fu S	2021	Not specific	SII	320-802	12	1.53 (1.27-1.83)	77%	(118)
Qiu Y	2021	Not specific	SII	320-802	8	1.40 (1.08-1.81)	88%	(119)
Yin J	2023	Surgery	CONUT	1-5	14	1.75 (1.55-1.96)	12%	(120)
Takagi K	2019	Surgery	CONUT	1-5	4	1.85 (1.38-2.48)	54%	(121)
Pang H	2024	Surgery	ALI	24.81-40.50	4	1.45 (1.02-1.73)	0%	(122)
Kim MR	2020	Not specific	CRP	0.3-13.9	11	1.65 (1.27-2.15)	86%	(91)

ALI, advanced lung cancer inflammation index; CONUT, controlling nutritional status; CRP, C-reactive protein; HR, hazard ratio; LMR, lymphocyte-to-monocyte ratio; NLR, neutrophil-to-lymphocyte ratio; OS, overall survival; PLR, platelet-to-lymphocyte ratio; PNI, prognostic nutritional index; SII, systemic immune-inflammation index.

**Table 2:** Randomized controlled studies and meta-analyses investigating strategies to preserve or enhance systemic immunity following radical gastrectomy.

Author	Years	Design	Intervention	Control	Findings	Reference
Ma Z	2016	RCT	LAG, 129	OG, 107	Higher CD3^+^, CD4^+^, CD4^+^/CD8^+^ cell ratios and lower IL-6, TNF and CRP in LAG group	(128)
Aoyama T	2014	RCT	LAG, 13	OG, 13	No significant difference between two groups in WBC count, IL-6 and CRP	(129)
Fujii K	2003	RCT	LAG, 10	OG, 10	Higher TNF-γ production, lower IL-4 production in LAG group	(130)
Shu ZB	2015	Meta-analysis	LAG, 427	OG, 240	LAG is associated with significantly lower serum IL-6 levels	(131)
Lv AQ	2022	RCT	Low anesthetic depth, 40	High anesthetic depth, 40	The perioperative release of inflammatory factors (IL-6, IL-10) is less in patients with low anesthetic depth	(132)
Liu W	2019	RCT	Epidural and general anesthesia, 54	General anesthesia, 53	Lower IL-1, IL-8, hs-CRP and TNF-α, while higher CD3^+^, CD4^+^ and CD4^+^/CD8^+^ cell ratio in the epidural and general anesthesia group	(133)
Wang L	2019	RCT	Epidural and general anesthesia, 20	General anesthesia, 20	CD3^+^ T cells decreased less, while IL-4 and IL-6 increased less in the epidural and general anesthesia group	(134)
Kun L	2014	RCT	Epidural and general anesthesia	General anesthesia	Less suppression of NK cell activity, higher IL-2 and IL-10, and lower IL-1β and IL-6 in the epidural and general anesthesia group	(135)
Konstantis G	2023	Meta-analysis	Epidural and general anesthesia, 54	General anesthesia, 53	Higher NK cells and CD4^+^ T cells in the epidural and general anesthesia group	(136)
Liu R	2019	RCT	Transversus abdominis plane, 30	General anesthesia, 31	IL-6 and IL-10 were significantly lower in the transversus abdominis plane group	(137)
Moon J	2023	RCT	Dexmedetomidine, 42	Control, 42	The IL-6 levels at the end of the surgery was significantly lower in the dexmedetomidine group	(138)
Zhu M	2021	RCT	Quadratus lumborum block, 32	Control, 32	HMGB1, TNF-α, and IL-6 were significantly decreased after surgery in the quadratus lumborum block group	(139)
Lao WL	2021	RCT	Oxycodone, 30	Sufentanil, 30	Lower postoperative IL-6 while higher IL-10 in the oxycodone group	(140)
Kim Y	2015	RCT	Esmolol, 26	Control, 32	Esmolol decreased postoperative IL-6, IL-10, IL-4 and CRP	(141)
Zang YF	2018	RCT	ERAS, 20	Control, 20	Lower WBC, CRP, IL-6 in the ERAS group	(142)
Tang A	2021	RCT	Goal-directed fluid therapy, 37	Conventional fluids, 37	Lower CPR, IL-6 and PCT in the goal-directed fluid therapy group	(143)
Miyachi T	2013	Surgery	Cystine and theanine, 15	Placebo, 18	Significantly lower IL-6, CRP, and neutrophils in the intervention group	(144)
Cao W	2022	RCT	Clostridium butyricum, 47	Placebo, 45	Significantly reduced leucocytes, neutrophils, IL-1β, IL-6, and TNF-α, markedly enhanced immunoglobulin and lymphocytes in the intervention group	(145)
Fu H	2022	Meta-analysis	Enteral immunonutrition, 505	Standard enteral nutrition, 551	Higher proalbumin, IgM, and IgG in the enteral immunonutrition group	(146)
Cheng Y	2018	Meta-analysis	Enteral immunonutrition, 297	Standard enteral nutrition, 286	Higher CD4^+^, CD4^+^/CD8^+^, IgM, IgG, and lymphocytes in the enteral immunonutrition group	(147)

CRP, C-reactive protein; ERAS, enhanced recovery after surgery; HMGB1, high mobility group box 1 protein; IL, interleukin; LAG, laparoscopic-assisted gastrectomy; NK, natural killer; OG, open gastrectomy; PCT, procalcitonin; RCT, randomized controlled study; TNF, tumor necrosis factor; WBC, white blood cell.

Nevertheless, despite tremendous interest in the development of predictive and prognostic biomarkers derived from peripheral immunity, no such biomarkers have shown sufficient ability to guide bedside decision-making. First, because of the wide range of cutoff values used across studies, the optimal values of these indices as prognostic factors are unknown and need to be standardized through multicenter and international studies. Second, systemic immunity is continuously influenced by various factors, including cancer treatment. Although changes in some indices during therapy have shown better prognostic value, studies dedicated to monitoring the dynamics of systemic immune biomarkers in GC are limited (15, 24). Finally, the predictive and prognostic values of systemic immune biomarkers may be context dependent. For example, the prognostic value of circulating cytokines was exclusive to patients receiving immunotherapy in combination with chemotherapy but not to patients receiving chemotherapy alone (15). Therefore, further studies are needed to fully understand why the prognostic value of systemic immune components in GC patients is inconsistent across different contexts.

### 4.5 Harnessing systemic immunity for GC therapy

Although the exact mechanisms underlying the contribution of systemic immunity to GC progression remain unknown, its distinct perturbations during carcinogenesis and prognostic value suggest that systemic immunity has the potential to be harnessed for GC therapy. As surgery and anesthesia are two of the strongest inducers of the stress response, studies have focused on exploring strategies to alleviate their effects on systemic immune function and inflammation (123). Compared with open gastrectomy, laparoscopic-assisted gastrectomy (LAG) has a weaker inflammatory response and less impact on the immune system. Furthermore, in recent years, the noninferiority of LAG in long-term survival has been strongly established for both early and locally advanced GC patients (124–127). Therefore, LAG is recommended for GC patients without contraindications to reduce the detrimental effects of surgery on systemic immunity. In terms of anesthesia and analgesia, the available evidence supports the combination of epidural analgesia and general anesthesia, which has the potential to improve systemic immunity while inhibiting the inflammatory response. As discussed above, activation of the sympathetic nervous system contributes the most to postoperative immune suppression; therefore, intraoperative administration of esmolol, a β-receptor blocker, decreases the inflammatory response and CRP production in a dose-dependent manner. In addition to these examples, various other perioperative strategies, including analgesics, anesthesia at low depths, goal-directed fluid therapy, enhanced recovery after surgery (ERAS), probiotics and enteral immunonutrition, have also shown beneficial effects on systemic immunity, and their regular administration is worthy of further exploration (**Table 2**) (128–147).

In addition to these strategies aimed at preserving systemic immunity through a reduction in the stress response and inflammation following radical gastrectomy, another means to harness systemic immunity for GC therapy is to directly enhance the anticancer response. One unsophisticated strategy involves the use of recombinant cytokines, such as IL-2, type I IFNs and granulocyte-macrophage colony-stimulating factor (GM-CSF), which can activate peripheral lymphocytes and improve survival (148–151). However, cytokine therapy has been discontinued in recent years, largely owing to severe systemic adverse events. Cell-based immunotherapies, such as cytokine-induced killer cell (CIK) therapy, dendritic cell-based vaccines, and chimeric antigen receptor (CAR) T or NK cell therapy, mainly involve obtaining therapeutic cells from the periphery and have shown promising results in both preclinical and clinical trials in GC (152–157). Another class of immunotherapy, ICIs, has revolutionized the field of oncology in the past decade. Many clinical trials have demonstrated the efficacy of ICIs in patients with GC (4–7). Although many studies have focused on the TME to investigate the mechanisms underlying the anticancer effects of ICIs, the involvement of systemic immunity has also been noted in recent years. As persistence in the TME rapidly drives dysfunctional differentiation of T cells, effective immunotherapies need continuous new effector T-cell infiltration. Several studies have shown that the *de novo* immune response mainly occurs in the periphery (158–160). In GC patients, ICIs have been shown to increase the levels of circulating IL-2 and IFN-γ and enhance the activation of central/effector memory and effector subsets of CD4+/CD8+ T cells (15, 161). As discussed above, traditional therapies for GC have detrimental effects on systemic immunity; therefore, pairing chemotherapy with ICIs significantly improved the oncological outcomes of GC patients with GC (4–7). In contrast, the application of immunotherapy during the perioperative timeframe has been limited by the established and theoretical risks pertinent around the time of surgery. However, as perioperative immune preservation or stimulation have various advantages, further investigations are needed to develop potential strategies.

## 5 Conclusions and future perspectives

With the progression of GC, not only the local but also the systemic immune landscape are strongly perturbed by cancer. In addition, traditional treatments for GC, including radical gastrectomy and chemotherapy, also have detrimental effects on systemic immunity. Although effector immune cells in the TME are the key executors, the localized anticancer immune response cannot persist without continuous communication with the periphery. As systemic immunity closely participates in the progression of GC and is a critical determinant of the efficacy of other therapeutic methods, systemic immunity can be widely applied as a biomarker for GC detection, prediction and prognosis and can be harnessed for GC treatment.

Nevertheless, the majority of currently available data are limited to exploring the perturbations and their associations with therapeutic efficacy and oncological outcomes, and few studies have elucidated the underlying mechanisms involved. Although some critical progress has been made in other cancer types, because systemic immune alterations vary across cancer tissue origins, disease stages and patient characteristics, more studies are needed to clarify the distinct immune states and critical mechanisms involved in directing treatment development to restore an anticancer immune macroenvironment. In recent years, the progress of single-cell technologies has provided many impressive transcriptomic, epigenomic and proteomic data on the immune microenvironment, which can also be applied to assess alterations in systemic immunity. In addition to traditional measurement methods, such as peripheral blood cell counts, circulating molecule detection and flow cytometry, these single-cell technologies can inform the distinct types of systemic immune organization in GC, which will aid personalized treatment designed to pair with traditional therapies to alleviate their detrimental effects on systemic immunity or to directly activate the anticancer response of systemic immunity. Although various strategies have been shown to improve the function of systemic immunity in GC patients during traditional therapy, the translation of these effects into survival benefits is limited, and further studies are needed to determine the underlying mechanisms involved. On the other hand, immunotherapy has achieved impressive success in patients with GC; however, the majority of patients obtained no benefit from this therapeutic strategy. Previous studies on the mechanisms underlying resistance to immunotherapy or biomarkers for patient stratification have focused mainly on the TME. Owing to the close associations with effective anticancer immune responses, strategies harnessing systemic immunity to improve the oncological outcomes of patients with GC warrant further research.

## References

[1] Zhang T Chen H Zhang Y Yin X Man J Yang X. Global changing trends in incidence and mortality of gastric cancer by age and sex, 1990-2019: Findings from Global Burden of Disease Study. J Cancer. (2021) 12:6695–705. doi: 10.7150/jca.62734 PMC8517992 34659559

[2] Bray F Laversanne M Sung H Ferlay J Siegel RL Soerjomataram I. Global cancer statistics 2022: GLOBOCAN estimates of incidence and mortality worldwide for 36 cancers in 185 countries. CA Cancer J Clin. (2024) 74:229–63. doi: 10.3322/caac.21834 38572751

[3] Joshi SS Badgwell BD. Current treatment and recent progress in gastric cancer. CA Cancer J Clin. (2021) 71:264–79. doi: 10.3322/caac.21657 PMC9927927 33592120

[4] Chen LT Satoh T Ryu MH Chao Y Kato K Chung HC. A phase 3 study of nivolumab in previously treated advanced gastric or gastroesophageal junction cancer (ATTRACTION-2): 2-year update data. Gastric Cancer. (2020) 23:510–9. doi: 10.1007/s10120-019-01034-7 PMC7165140 31863227

[5] Shitara K Van Cutsem E Bang YJ Fuchs C Wyrwicz L Lee KW. Efficacy and safety of pembrolizumab or pembrolizumab plus chemotherapy vs chemotherapy alone for patients with first-line, advanced gastric cancer: the KEYNOTE-062 phase 3 randomized clinical trial. JAMA Oncol. (2020) 6:1571–80. doi: 10.1001/jamaoncol.2020.3370 PMC7489405 32880601

[6] Shitara K Ajani JA Moehler M Garrido M Gallardo C Shen L. Nivolumab plus chemotherapy or ipilimumab in gastro-oesophageal cancer. Nature. (2022) 603:942–8. doi: 10.1038/s41586-022-04508-4 PMC8967713 35322232

[7] Janjigian YY Kawazoe A Yañez P Li N Lonardi S Kolesnik O. The KEYNOTE-811 trial of dual PD-1 and HER2 blockade in HER2-positive gastric cancer. Nature. (2021) 600:727–30. doi: 10.1038/s41586-021-04161-3 PMC8959470 34912120

[8] Kono K Nakajima S Mimura K. Biomarker-oriented chemo-immunotherapy for advanced gastric cancer. Int J Clin Oncol. (2024) 29:865–72. doi: 10.1007/s10147-024-02525-z 38647874

[9] Mou P Ge QH Sheng R Zhu TF Liu Y Ding K. Research progress on the immune microenvironment and immunotherapy in gastric cancer. Front Immunol. (2023) 14:1291117. doi: 10.3389/fimmu.2023.1291117 38077373 PMC10701536

[10] Hiam-Galvez KJ Allen BM Spitzer MH. Systemic immunity in cancer. Nat Rev Cancer. (2021) 21:345–59. doi: 10.1038/s41568-021-00347-z PMC8034277 33837297

[11] Gabrilovich DI Ostrand-Rosenberg S Bronte V. Coordinated regulation of myeloid cells by tumours. Nat Rev Immunol. (2012) 12:253–68. doi: 10.1038/nri3175 PMC3587148 22437938

[12] Allen BM Hiam KJ Burnett CE Venida A DeBarge R Tenvooren I. Systemic dysfunction and plasticity of the immune macroenvironment in cancer models. Nat Med. (2020) 26:1125–34. doi: 10.1038/s41591-020-0892-6 PMC7384250 32451499

[13] Propper DJ Balkwill FR. Harnessing cytokines and chemokines for cancer therapy. Nat Rev Clin Oncol. (2022) 19:237–53. doi: 10.1038/s41571-021-00588-9 34997230

[14] Yang E Chua W Ng W Roberts TL. Peripheral cytokine levels as a prognostic indicator in gastric cancer: A review of existing literature. Biomedicines. (2021) 9:1916. doi: 10.3390/biomedicines9121916 34944729 PMC8698340

[15] Hou Y Li X Yang Y Shi H Wang S Gao M. Serum cytokines and neutrophil-to-lymphocyte ratio as predictive biomarkers of benefit from PD-1 inhibitors in gastric cancer. Front Immunol. (2023) 14:1274431. doi: 10.3389/fimmu.2023.1274431 38022654 PMC10643875

[16] Sánchez-Zauco N Torres J Gómez A Camorlinga-Ponce M Muñoz-Pérez L Herrera-Goepfert R. Circulating blood levels of IL-6, IFN-γ, and IL-10 as potential diagnostic biomarkers in gastric cancer: a controlled study. BMC Cancer. (2017) 17:384. doi: 10.1186/s12885-017-3310-9 28558708 PMC5450104

[17] Lippitz BE. Cytokine patterns in patients with cancer: a systematic review. Lancet Oncol. (2013) 14:e218–28. doi: 10.1016/S1470-2045(12)70582-X 23639322

[18] Ashizawa T Okada R Suzuki Y Takagi M Yamazaki T Sumi T. Clinical significance of interleukin-6 (IL-6) in the spread of gastric cancer: role of IL-6 as a prognostic factor. Gastric Cancer. (2005) 8:124–31. doi: 10.1007/s10120-005-0315-x 15864720

[19] Tonouchi H Miki C Tanaka K Kusunoki M. Profile of monocyte chemoattractant protein-1 circulating levels in gastric cancer patients. Scand J Gastroenterol. (2002) 37:830–3. doi: 10.1080/gas.37.7.830.833 12190098

[20] Amedei A Della Bella C Silvestri E Prisco D D'Elios MM. T cells in gastric cancer: friends or foes. Clin Dev Immunol. (2012) 2012:690571. doi: 10.1155/2012/690571 22693525 PMC3369415

[21] Szkaradkiewicz A Karpiński TM Drews M Borejsza-Wysocki M Majewski P Andrzejewska E. Natural killer cell cytotoxicity and immunosuppressive cytokines (IL-10, TGF-beta1) in patients with gastric cancer. J BioMed Biotechnol. (2010) 2010:901564. doi: 10.1155/2010/901564 20445748 PMC2860365

[22] Jiang P Zhang Y Ru B Yang Y Vu T Paul R. Systematic investigation of cytokine signaling activity at the tissue and single-cell levels. Nat Methods. (2021) 18:1181–91. doi: 10.1038/s41592-021-01274-5 PMC8493809 34594031

[23] Cárdenas DM Sánchez AC Rosas DA Rivero E Paparoni MD Cruz MA. Preliminary analysis of single-nucleotide polymorphisms in IL-10, IL-4, and IL-4Rα genes and profile of circulating cytokines in patients with gastric Cancer. BMC Gastroenterol. (2018) 18:184. doi: 10.1186/s12876-018-0913-9 30526523 PMC6288868

[24] Qi Q Peng Y Zhu M Zhang Y Bao Y Zhang X. Association between serum levels of 12 different cytokines and short-term efficacy of anti-PD-1 monoclonal antibody combined with chemotherapy in advanced gastric cancer. Int Immunopharmacol. (2023) 114:109553. doi: 10.1016/j.intimp.2022.109553 36516540

[25] Guo GH Xie YB Zhang PJ Jiang T. Blood index panel for gastric cancer detection. World J Gastrointest Surg. (2022) 14:1026–36. doi: 10.4240/wjgs.v14.i9.1026 PMC9521474 36185564

[26] Epplein M Xiang YB Cai Q Peek RM Jr Li H Correa P. Circulating cytokines and gastric cancer risk. Cancer Causes Control. (2013) 24:2245–50. doi: 10.1007/s10552-013-0284-z PMC3828746 24052422

[27] Zou Z Zhao L Su S Liu Q Yu L Wei J. The plasma levels of 12 cytokines and growth factors in patients with gastric cancer. Med (Baltimore). (2018) 97:e0413. doi: 10.1097/MD.0000000000010413 PMC5959396 29742685

[28] Liang J Li Y Liu X Xu X Zhao Y. Relationship between cytokine levels and clinical classification of gastric cancer. Asian Pac J Cancer Prev. (2011) 12:1803–6. 22126569

[29] Wang TT Zhao YL Peng LS Chen N Chen W Lv YP. Tumour-activated neutrophils in gastric cancer foster immune suppression and disease progression through GM-CSF-PD-L1 pathway. Gut. (2017) 66:1900–11. doi: 10.1136/gutjnl-2016-313075 PMC5739867 28274999

[30] Chen J Liu Z Gao G Mo Y Zhou H Huang W. Efficacy of circulating microRNA-130b and blood routine parameters in the early diagnosis of gastric cancer. Oncol Lett. (2021) 22:725. doi: 10.3892/ol.2021.12986 34429765 PMC8371962

[31] Arii K Tanimura H Iwahashi M Tsunoda T Tani M Noguchi K. Neutrophil functions and cytokine production in patients with gastric cancer. Hepatogastroenterology. (2000) 47:291–7. 10690623

[32] Mao FY Zhao YL Lv YP Teng YS Kong H Liu YG. CD45(+)CD33(low)CD11b(dim) myeloid-derived suppressor cells suppress CD8(+) T cell activity via the IL-6/IL-8-arginase I axis in human gastric cancer. Cell Death Dis. (2018) 9:763. doi: 10.1038/s41419-018-0803-7 29988030 PMC6037756

[33] Ross DS Neer RM Ridgway EC Daniels GH. Subclinical hyperthyroidism and reduced bone density as a possible result of prolonged suppression of the pituitary-thyroid axis with L-thyroxine. Am J Med. (1987) 82:1167–70. doi: 10.1016/0002-9343(87)90219-1 3605133

[34] Zhu T Zou X Yang C Li L Wang B Li R. Neutrophil extracellular traps promote gastric cancer metastasis by inducing epithelial−mesenchymal transition. Int J Mol Med. (2021) 48:127 [pii]. doi: 10.3892/ijmm.2021.4960 34013374 PMC8128417

[35] Yamane T Sakita M Kasuga M Nishioka B Fujita Y Majima S. Monocyte count, monocyte chemotaxis and chemotactic factor inactivator in gastric cancer patients. Jpn J Surg. (1981) 11:422–7. doi: 10.1007/BF02469025 7328934

[36] Wang Z Yin N Zhang Z Zhang Y Zhang G Chen W. Upregulation of T-cell immunoglobulin and mucin-domain containing-3 (Tim-3) in monocytes/macrophages associates with gastric cancer progression. Immunol Invest. (2017) 46:134–48. doi: 10.1080/08820139.2016.1229790 27911104

[37] Gardner A Ruffell B. Dendritic cells and cancer immunity. Trends Immunol. (2016) 37:855–65. doi: 10.1016/j.it.2016.09.006 PMC5135568 27793569

[38] Chen J Yang J Jiang J Zhuang Y He W. Function and subsets of dendritic cells and natural killer cells were decreased in gastric cancer. Int J Clin Exp Pathol. (2014) 7:8304–11. PMC4270606 25550889

[39] Chang LL Wang SW Wu IC Yu FJ Su YC Chen YP. Impaired dendritic cell maturation and IL-10 production following H. pylori stimulation in gastric cancer patients. Appl Microbiol Biotechnol. (2012) 96:211–20. doi: 10.1007/s00253-012-4034-z PMC3433674 22526791

[40] Coutant F Miossec P. Altered dendritic cell functions in autoimmune diseases: distinct and overlapping profiles. Nat Rev Rheumatol. (2016) 12:703–15. doi: 10.1038/nrrheum.2016.147 27652503

[41] Xu DP Shi WW Zhang TT Lv HY Li JB Lin A. Elevation of HLA-G-expressing DC-10 cells in patients with gastric cancer. Hum Immunol. (2016) 77:800–4. doi: 10.1016/j.humimm.2016.01.003 26773190

[42] Yu H Mei Y Dong Y Chen C Lin X Jin H. CCR9-CCL25 mediated plasmacytoid dendritic cell homing and contributed the immunosuppressive microenvironment in gastric cancer. Transl Oncol. (2023) 33:101682. doi: 10.1016/j.tranon.2023.101682 37126939 PMC10172990

[43] Huang XM Liu XS Lin XK Yu H Sun JY Liu XK. Role of plasmacytoid dendritic cells and inducible costimulator-positive regulatory T cells in the immunosuppression microenvironment of gastric cancer. Cancer Sci. (2014) 105:150–8. doi: 10.1111/cas.12327 PMC4317822 24261990

[44] Li J Chen Z Li Q Liu R Zheng J Gu Q. Study of miRNA and lymphocyte subsets as potential biomarkers for the diagnosis and prognosis of gastric cancer. PeerJ. (2024) 12:e16660. doi: 10.7717/peerj.16660 38259671 PMC10802158

[45] Xu J Guo R Jia J He Y He S. Activation of Toll-like receptor 2 enhances peripheral and tumor-infiltrating CD8(+) T cell cytotoxicity in patients with gastric cancer. BMC Immunol. (2021) 22:67. doi: 10.1186/s12865-021-00459-z 34620075 PMC8499526

[46] Zhong C Song Z Li M. Gastric cancer patients display a distinctive population of IFNg(+)IL10(+) double positive CD8 T cells, which persists longer during prolonged activation. Exp Cell Res. (2019) 382:111487. doi: 10.1016/j.yexcr.2019.06.032 31260655 PMC7094396

[47] Takano S Saito H Ikeguchi M. An increased number of PD-1+ and Tim-3+ CD8+ T cells is involved in immune evasion in gastric cancer. Surg Today. (2016) 46:1341–7. doi: 10.1007/s00595-016-1305-9 26801344

[48] Zhong F Cui D Tao H Du H Xing C. IL-17A-producing T cells and associated cytokines are involved in the progression of gastric cancer. Oncol Rep. (2015) 34:2365–74. doi: 10.3892/or.2015.4246 26352729

[49] Zhuang Y Peng LS Zhao YL Shi Y Mao XH Chen W. CD8(+) T cells that produce interleukin-17 regulate myeloid-derived suppressor cells and are associated with survival time of patients with gastric cancer. Gastroenterology. (2012) 143:951–62.e8. doi: 10.1053/j.gastro.2012.06.010 22710190

[50] Li H Cao GM Gu GL Li SY Yan Y Fu Z. Expression characteristics of peripheral lymphocyte programmed death 1 and FoxP3(+) Tregs in gastric cancer during surgery and chemotherapy. World J Gastroenterol. (2023) 29:5582–92. doi: 10.3748/wjg.v29.i40.5582 PMC10642441 37970473

[51] Zhang Y Wu J Zhang H Wu C. The regulation between CD4(+)CXCR5(+) follicular helper T (Tfh) cells and CD19(+)CD24(hi)CD38(hi) regulatory B (Breg) cells in gastric cancer. J Immunol Res. (2022) 2022:9003902. doi: 10.1155/2022/9003902 36339942 PMC9629923

[52] Wang WW Yuan XL Chen H Xie GH Ma YH Zheng YX. CD19+CD24hiCD38hiBregs involved in downregulate helper T cells and upregulate regulatory T cells in gastric cancer. Oncotarget. (2015) 6:33486–99. doi: 10.18632/oncotarget.5588 PMC4741780 26378021

[53] Shimasaki N Jain A Campana D. NK cells for cancer immunotherapy. Nat Rev Drug Discovery. (2020) 19:200–18. doi: 10.1038/s41573-019-0052-1 31907401

[54] Han B Mao FY Zhao YL Lv YP Teng YS Duan M. Altered NKp30, NKp46, NKG2D, and DNAM-1 expression on circulating NK cells is associated with tumor progression in human gastric cancer. J Immunol Res. (2018) 2018:6248590. doi: 10.1155/2018/6248590 30255106 PMC6140275

[55] Wang Z Zhu J Gu H Yuan Y Zhang B Zhu D. The clinical significance of abnormal Tim-3 expression on NK cells from patients with gastric cancer. Immunol Invest. (2015) 44:578–89. doi: 10.3109/08820139.2015.1052145 26214042

[56] Peng YP Zhu Y Zhang JJ Xu ZK Qian ZY Dai CC. Comprehensive analysis of the percentage of surface receptors and cytotoxic granules positive natural killer cells in patients with pancreatic cancer, gastric cancer, and colorectal cancer. J Transl Med. (2013) 11:262. doi: 10.1186/1479-5876-11-262 24138752 PMC3854023

[57] de la Torre JC Goldsmith HS. Increased blood flow enhances axon regeneration after spinal transection. Neurosci Lett. (1988) 94:269–73. doi: 10.1016/0304-3940(88)90029-8 2905029

[58] Lindgren Å Yun CH Sjöling Å Berggren C Sun JB Jonsson E. Impaired IFN-γ production after stimulation with bacterial components by natural killer cells from gastric cancer patients. Exp Cell Res. (2011) 317:849–58. doi: 10.1016/j.yexcr.2011.01.006 21255568

[59] Zhang J Zhang L Duan S Li Z Li G Yu H. Single and combined use of the platelet-lymphocyte ratio, neutrophil-lymphocyte ratio, and systemic immune-inflammation index in gastric cancer diagnosis. Front Oncol. (2023) 13:1143154. doi: 10.3389/fonc.2023.1143154 37064093 PMC10098186

[60] Uzunoglu H Kaya S. Does systemic immune inflammation index have predictive value in gastric cancer prognosis. North Clin Istanb. (2023) 10:24–32. doi: 10.14744/nci.2021.71324 36910431 PMC9996656

[61] Zhao Q Dong L Liang H Pang K Wang P Ge R. Evaluation of multiple biological indicators for combined diagnosis of gastric cancer: A retrospective analysis. Med (Baltimore). (2022) 101:e31904. doi: 10.1097/MD.0000000000031878 PMC9704904 36451446

[62] Servis D Busic Z Stipancic I Patrlj L Gagro A. Serum cytokine changes after gastric resection or gastrectomy for gastric cancer. Hepatogastroenterology. (2008) 55:1868–72. 19102411

[63] Sun HL Dong YC Wang CQ Qian YN Wang ZY. Effects of postoperative analgesia with the combination of tramadol and lornoxicam on serum inflammatory cytokines in patients with gastric cancer. Int J Clin Pharmacol Ther. (2014) 52:1023–9. doi: 10.5414/CP202190 25295719

[64] Okuno K Tanaka A Shigeoka H Hirai N Kawai I Kitano Y. Suppression of T-cell function in gastric cancer patients after total gastrectomy with splenectomy: implications of splenic autotransplantation. Gastric Cancer. (1999) 2:20–5. doi: 10.1007/s101200050016 11957066

[65] Zhi X Kuang X Li J. The impact of perioperative events on cancer recurrence and metastasis in patients after radical gastrectomy: A review. Cancers (Basel). (2022) 14:3496. doi: 10.3390/cancers14143496 35884557 PMC9319233

[66] Hiller JG Perry NJ Poulogiannis G Riedel B Sloan EK. Perioperative events influence cancer recurrence risk after surgery. Nat Rev Clin Oncol. (2018) 15:205–18. doi: 10.1038/nrclinonc.2017.194 29283170

[67] Tai LH de Souza CT Bélanger S Ly L Alkayyal AA Zhang J. Preventing postoperative metastatic disease by inhibiting surgery-induced dysfunction in natural killer cells. Cancer Res. (2013) 73:97–107. doi: 10.1158/0008-5472.CAN-12-1993 23090117

[68] Gryglewski A Majcher P Bryniarski K Konturek S Ptak M Ptak W. Mesenteric lymph node Tgammadelta cells induced by gastrectomy in mice suppress cell-mediated immune response *in vitro* via released TGF-beta. J Surg Res. (2007) 142:66–71. doi: 10.1016/j.jss.2006.10.050 17612560

[69] Bosiljcic M Cederberg RA Hamilton MJ LePard NE Harbourne BT Collier JL. Targeting myeloid-derived suppressor cells in combination with primary mammary tumor resection reduces metastatic growth in the lungs. Breast Cancer Res. (2019) 21:103. doi: 10.1186/s13058-019-1189-x 31488209 PMC6727565

[70] Shaked Y. The pro-tumorigenic host response to cancer therapies. Nat Rev Cancer. (2019) 19:667–85. doi: 10.1038/s41568-019-0209-6 31645711

[71] Zhang M Fan Y Che X Hou K Zhang C Li C. 5-FU-induced upregulation of exosomal PD-L1 causes immunosuppression in advanced gastric cancer patients. Front Oncol. (2020) 10:492. doi: 10.3389/fonc.2020.00492 32391259 PMC7188923

[72] Zhang F Li R Yang Y Shi C Shen Y Lu C. Specific decrease in B-cell-derived extracellular vesicles enhances post-chemotherapeutic CD8(+) T cell responses. Immunity. (2019) 50:738–50.e7. doi: 10.1016/j.immuni.2019.01.010 30770248

[73] Bruchard M Mignot G Derangère V Chalmin F Chevriaux A Végran F. Chemotherapy-triggered cathepsin B release in myeloid-derived suppressor cells activates the Nlrp3 inflammasome and promotes tumor growth. Nat Med. (2013) 19:57–64. doi: 10.1038/nm.2999 23202296

[74] Takeuchi S Baghdadi M Tsuchikawa T Wada H Nakamura T Abe H. Chemotherapy-derived inflammatory responses accelerate the formation of immunosuppressive myeloid cells in the tissue microenvironment of human pancreatic cancer. Cancer Res. (2015) 75:2629–40. doi: 10.1158/0008-5472.CAN-14-2921 25952647

[75] Schreiber RD Old LJ Smyth MJ. Cancer immunoediting: integrating immunity's roles in cancer suppression and promotion. Science. (2011) 331:1565–70. doi: 10.1126/science.1203486 21436444

[76] Garner H de Visser KE. Immune crosstalk in cancer progression and metastatic spread: a complex conversation. Nat Rev Immunol. (2020) 20:483–97. doi: 10.1038/s41577-019-0271-z 32024984

[77] Spitzer MH Carmi Y Reticker-Flynn NE Kwek SS Madhireddy D Martins MM. Systemic immunity is required for effective cancer immunotherapy. Cell. (2017) 168:487–502.e15. doi: 10.1016/j.cell.2016.12.022 28111070 PMC5312823

[78] Fransen MF Schoonderwoerd M Knopf P Camps MG Hawinkels LJ Kneilling M. Tumor-draining lymph nodes are pivotal in PD-1/PD-L1 checkpoint therapy. JCI Insight. (2018) 3:e124507. doi: 10.1172/jci.insight.124507 30518694 PMC6328025

[79] Ruan DY Chen YX Wei XL Wang YN Wang ZX Wu HX. Elevated peripheral blood neutrophil-to-lymphocyte ratio is associated with an immunosuppressive tumour microenvironment and decreased benefit of PD-1 antibody in advanced gastric cancer. Gastroenterol Rep (Oxf). (2021) 9:560–70. doi: 10.1093/gastro/goab032 PMC8677531 34925853

[80] Peinado H Zhang H Matei IR Costa-Silva B Hoshino A Rodrigues G. Pre-metastatic niches: organ-specific homes for metastases. Nat Rev Cancer. (2017) 17:302–17. doi: 10.1038/nrc.2017.6 28303905

[81] Hedrick CC Malanchi I. Neutrophils in cancer: heterogeneous and multifaceted. Nat Rev Immunol. (2022) 22:173–87. doi: 10.1038/s41577-021-00571-6 34230649

[82] Gaertner F Massberg S. Patrolling the vascular borders: platelets in immunity to infection and cancer. Nat Rev Immunol. (2019) 19:747–60. doi: 10.1038/s41577-019-0202-z 31409920

[83] Ge X Zhang X Ma Y Chen S Chen Z Li M. Diagnostic value of macrophage inhibitory cytokine 1 as a novel prognostic biomarkers for early gastric cancer screening. J Clin Lab Anal. (2021) 35:e23568. doi: 10.1002/jcla.23568 32918498 PMC7843257

[84] Li J Xu L Run ZC Feng W Liu W Zhang PJ. Multiple cytokine profiling in serum for early detection of gastric cancer. World J Gastroenterol. (2018) 24:2269–78. doi: 10.3748/wjg.v24.i21.2269 PMC5989241 29881236

[85] Fang T Wang Y Yin X Zhai Z Zhang Y Yang Y. Diagnostic sensitivity of NLR and PLR in early diagnosis of gastric cancer. J Immunol Res. (2020) 2020:9146042. doi: 10.1155/2020/9146042 32211444 PMC7081040

[86] Nakamura N Kinami S Fujii Y Miura S Fujita J Kaida D. The neutrophil/lymphocyte ratio as a predictor of peritoneal metastasis during staging laparoscopy for advanced gastric cancer: a retrospective cohort analysis. World J Surg Oncol. (2019) 17:108. doi: 10.1186/s12957-019-1651-3 31238937 PMC6593512

[87] Li J Zhang Y Hu DM Gong TP Xu R Gao J. Impact of postoperative complications on long-term outcomes of patients following surgery for gastric cancer: A systematic review and meta-analysis of 64 follow-up studies. Asian J Surg. (2020) 43:719–29. doi: 10.1016/j.asjsur.2019.10.007 31703889

[88] Dan Zeng CD Tong YX Xiao AT Gao C Zhang S. Peripheral lymphocyte subsets absolute counts as feasible clinical markers for predicting surgical outcome in gastric cancer patients after laparoscopic D2 gastrectomy: A prospective cohort study. J Inflammation Res. (2021) 14:5633–46. doi: 10.2147/JIR.S335847 PMC8565983 34744447

[89] Gülmez S Senger A Uzun O Ozduman O Ofluoglu C Subasi İ. Comparative analysis of preoperative ratio based markers in predicting postoperative infectious complications after gastrectomy. Pol Przegl Chir. (2022) 95:1–5. doi: 10.5604/01.3001.0015.9662 36807098

[90] Hwang SH Kim DJ. Nomogram for predicting infectious complications following curative gastrectomy using clinical and laboratory parameters. Anticancer Res. (2024) 44:1781–90. doi: 10.21873/anticanres.16978 38537986

[91] Peng X Zeng W Tang B He A Zhang M Luo R. Utility of pretreatment blood platelet-to-lymphocyte ratio in prediction of clinical outcomes and chemosensitivity in patients with advanced gastric cancer: A meta-analysis. Med Sci Monit. (2022) 28:e933449. doi: 10.12659/MSM.933449 35095093 PMC8815280

[92] Liu J Mao Y Mao C Wang D Dong L Zhu W. An on-treatment decreased trend of serum IL-6 and IL-8 as predictive markers quickly reflects short-term efficacy of PD-1 blockade immunochemotherapy in patients with advanced gastric cancer. J Immunol Res. (2024) 2024:3604935. doi: 10.1155/2024/3604935 38774604 PMC11108694

[93] Lee CK Lee JB Park SJ Che J Kwon WS Kim HS. Second-line chemoimmunotherapy with nivolumab and paclitaxel in immune-related biomarker-enriched advanced gastric cancer: a multicenter phase Ib/II study. Gastric Cancer. (2024) 27:118–30. doi: 10.1007/s10120-023-01435-9 37906316

[94] Ohe Y Fushida S Yamaguchi T Kinoshita J Saito H Okamoto K. Peripheral blood platelet-lymphocyte ratio is good predictor of chemosensitivity and prognosis in gastric cancer patients. Cancer Manag Res. (2020) 12:1303–11. doi: 10.2147/CMAR.S241069 PMC7039245 32110104

[95] He XX Du B Wu T Shen H. Prognostic analysis of related factors of adverse reactions to immunotherapy in advanced gastric cancer and establishment of a nomogram model. World J Gastrointest Oncol. (2024) 16:1268–80. doi: 10.4251/wjgo.v16.i4.1268 PMC11037037 38660670

[96] Szaflarska A Szczepanik A Siedlar M Czupryna A Sierzega M Popiela T. Preoperative plasma level of IL-10 but not of proinflammatory cytokines is an independent prognostic factor in patients with gastric cancer. Anticancer Res. (2009) 29:5005–12. 20044609

[97] Kim MR Kim AS Choi HI Jung JH Park JY Ko HJ. Inflammatory markers for predicting overall survival in gastric cancer patients: A systematic review and meta-analysis. PloS One. (2020) 15:e0236445. doi: 10.1371/journal.pone.0236445 32716955 PMC7384660

[98] Liu X Yu H Yan C Mei Y Lin C Hong Y. Plasmacytoid dendritic cells and ICOS(+) regulatory T cells predict poor prognosis in gastric cancer: A pilot study. J Cancer. (2019) 10:6711–5. doi: 10.7150/jca.34826 PMC6856898 31777600

[99] Szor DJ Dias AR Pereira MA Ramos M Zilberstein B Cecconello I. Prognostic role of neutrophil/lymphocyte ratio in resected gastric cancer: A systematic review and meta-analysis. Clinics (Sao Paulo). (2018) 73:e360. doi: 10.6061/clinics/2018/e360 29924187 PMC5996440

[100] Mellor KL Powell A Lewis WG. Systematic review and meta-analysis of the prognostic significance of neutrophil-lymphocyte ratio (NLR) after R0 gastrectomy for cancer. J Gastrointest Cancer. (2018) 49:237–44. doi: 10.1007/s12029-018-0127-y PMC6061213 29949048

[101] Li LL Pan LS. Prognostic value of neutrophil-to-lymphocyte ratio in gastric cancer patients treated with immune checkpoint inhibitors: A meta-analysis. Kaohsiung J Med Sci. (2023) 39:842–52. doi: 10.1002/kjm2.12694 PMC11895980 37166079

[102] Zhang S Qiu C Yu H Xu Y Xu X. Prognostic value of neutrophil to lymphocyte ratio in gastric cancer patients receiving immune checkpoint inhibitors: a systematic review and meta-analysis. Front Oncol. (2023) 13:1070019. doi: 10.3389/fonc.2023.1070019 37143942 PMC10153754

[103] Matsas S Aguiar PN Jr Del Giglio A. Neutrophil-to-lymphocyte ratio and platelet-to-lymphocyte ratio as biomarkers to prognosticate survival in advanced gastric cancer patients in the era of immunotherapy: a systematic review and meta-analysis. J Gastrointest Oncol. (2024) 15:33–51. doi: 10.21037/jgo-23-808 38482212 PMC10932683

[104] Du S Fang Z Ye L Sun H Deng G Wu W. Pretreatment neutrophil-to-lymphocyte ratio predicts the benefit of gastric cancer patients with systemic therapy. Aging (Albany NY). (2021) 13:17638–54. doi: 10.18632/aging.203256 PMC8312446 34245559

[105] Sun J Chen X Gao P Song Y Huang X Yang Y. Can the neutrophil to lymphocyte ratio be used to determine gastric cancer treatment outcomes? A systematic review and meta-analysis. Dis Markers. (2016) 2016:7862469. doi: 10.1155/2016/7862469 26924872 PMC4746375

[106] Xu Z Xu W Cheng H Shen W Ying J Cheng F. The prognostic role of the platelet-lymphocytes ratio in gastric cancer: A meta-analysis. PloS One. (2016) 11:e0163719. doi: 10.1371/journal.pone.0163719 27684077 PMC5042439

[107] Chen J Hong D Zhai Y Shen P. Meta-analysis of associations between neutrophil-to-lymphocyte ratio and prognosis of gastric cancer. World J Surg Oncol. (2015) 13:122. doi: 10.1186/s12957-015-0530-9 25889889 PMC4379945

[108] Hu G Wang S Wang S Huang L. Elevated baseline circulating platelet-to-lymphocyte ratio and survival in initial stage IV gastric cancer patients: A meta-analysis. PloS One. (2022) 17:e0265897. doi: 10.1371/journal.pone.0265897 35436305 PMC9015147

[109] Zhang X Zhang W Feng LJ. Prognostic significance of neutrophil lymphocyte ratio in patients with gastric cancer: a meta-analysis. PloS One. (2014) 9:e111906. doi: 10.1371/journal.pone.0111906 25401500 PMC4234250

[110] Zhang X Zhao W Yu Y Qi X Song L Zhang C. Clinicopathological and prognostic significance of platelet-lymphocyte ratio (PLR) in gastric cancer: an updated meta-analysis. World J Surg Oncol. (2020) 18:191. doi: 10.1186/s12957-020-01952-2 32731872 PMC7391520

[111] Cao W Yao X Cen D Zhi Y Zhu N Xu L. The prognostic role of platelet-to-lymphocyte ratio on overall survival in gastric cancer: a systematic review and meta-analysis. BMC Gastroenterol. (2020) 20:16. doi: 10.1186/s12876-020-1167-x 31959103 PMC6971934

[112] Gu X Gao XS Cui M Xie M Peng C Bai Y. Clinicopathological and prognostic significance of platelet to lymphocyte ratio in patients with gastric cancer. Oncotarget. (2016) 7:49878–87. doi: 10.18632/oncotarget.10490 PMC5226554 27409665

[113] Ma JY Liu Q. Clinicopathological and prognostic significance of lymphocyte to monocyte ratio in patients with gastric cancer: A meta-analysis. Int J Surg. (2018) 50:67–71. doi: 10.1016/j.ijsu.2018.01.002 29329786

[114] Mei P Feng W Zhan Y Guo X. Prognostic value of lymphocyte-to-monocyte ratio in gastric cancer patients treated with immune checkpoint inhibitors: a systematic review and meta-analysis. Front Immunol. (2023) 14:1321584. doi: 10.3389/fimmu.2023.1321584 38090560 PMC10711042

[115] Yang Y Gao P Song Y Sun J Chen X Zhao J. The prognostic nutritional index is a predictive indicator of prognosis and postoperative complications in gastric cancer: A meta-analysis. Eur J Surg Oncol. (2016) 42:1176–82. doi: 10.1016/j.ejso.2016.05.029 27293109

[116] Li J Xu R Hu DM Zhang Y Gong TP Wu XL. Prognostic nutritional index predicts outcomes of patients after gastrectomy for cancer: A systematic review and meta-analysis of nonrandomized studies. Nutr Cancer. (2019) 71:557–68. doi: 10.1080/01635581.2019.1577986 30793968

[117] Yang X Wu C. Systemic immune inflammation index and gastric cancer prognosis: A systematic review and meta−analysis. Exp Ther Med. (2024) 27:122. doi: 10.3892/etm.2024.12410 38410191 PMC10895464

[118] Fu S Yan J Tan Y Liu D. Prognostic value of systemic immune-inflammatory index in survival outcome in gastric cancer: a meta-analysis. J Gastrointest Oncol. (2021) 12:344–54. doi: 10.21037/jgo-20-252 PMC8107617 34012630

[119] Qiu Y Zhang Z Chen Y. Prognostic value of pretreatment systemic immune-inflammation index in gastric cancer: A meta-analysis. Front Oncol. (2021) 11:537140. doi: 10.3389/fonc.2021.537140 33777726 PMC7990885

[120] Yin J Qu J Liang X Wang M. Prognostic significance of controlling nutritional status score for patients with gastric cancer: A systematic review and meta−analysis. Exp Ther Med. (2023) 25:202. doi: 10.3892/etm.2023.11901 37090072 PMC10119667

[121] Takagi K Domagala P Polak WG Buettner S Wijnhoven B Ijzermans J. Prognostic significance of the controlling nutritional status (CONUT) score in patients undergoing gastrectomy for gastric cancer: a systematic review and meta-analysis. BMC Surg. (2019) 19:129. doi: 10.1186/s12893-019-0593-6 31488105 PMC6729085

[122] Pang H Dai L Chen L Chen X Chen Z Zhang S. Prognostic value of the advanced lung cancer inflammation index in patients with gastric cancer after radical gastrectomy: a propensity-score matching cohort study and meta-analysis. BMC Cancer. (2024) 24:583. doi: 10.1186/s12885-024-12349-9 38741082 PMC11089784

[123] Liu LB Li J Lai JX Shi S. Harnessing interventions during the immediate perioperative period to improve the long-term survival of patients following radical gastrectomy. World J Gastrointest Surg. (2023) 15:520–33. doi: 10.4240/wjgs.v15.i4.520 PMC10190732 37206066

[124] Katai H Mizusawa J Katayama H Morita S Yamada T Bando E. Survival outcomes after laparoscopy-assisted distal gastrectomy versus open distal gastrectomy with nodal dissection for clinical stage IA or IB gastric cancer (JCOG0912): a multicentre, non-inferiority, phase 3 randomised controlled trial. Lancet Gastroenterol Hepatol. (2020) 5:142–51. doi: 10.1016/S2468-1253(19)30332-2 31757656

[125] Kim HH Han SU Kim MC Kim W Lee HJ Ryu SW. Effect of laparoscopic distal gastrectomy vs open distal gastrectomy on long-term survival among patients with stage I gastric cancer: the KLASS-01 randomized clinical trial. JAMA Oncol. (2019) 5:506–13. doi: 10.1001/jamaoncol.2018.6727 PMC6459124 30730546

[126] Yu J Huang C Sun Y Su X Cao H Hu J. Effect of laparoscopic vs open distal gastrectomy on 3-year disease-free survival in patients with locally advanced gastric cancer: the CLASS-01 randomized clinical trial. JAMA. (2019) 321:1983–92. doi: 10.1001/jama.2019.5359 PMC6547120 31135850

[127] Hyung WJ Yang HK Park YK Lee HJ An JY Kim W. Long-term outcomes of laparoscopic distal gastrectomy for locally advanced gastric cancer: the KLASS-02-RCT randomized clinical trial. J Clin Oncol. (2020) 38:3304–13. doi: 10.1200/JCO.20.01210 32816629

[128] Ma Z Bao X Gu J. Effects of laparoscopic radical gastrectomy and the influence on immune function and inflammatory factors. Exp Ther Med. (2016) 12:983–6. doi: 10.3892/etm.2016.3404 PMC4950086 27446308

[129] Aoyama T Yoshikawa T Hayashi T Hasegawa S Tsuchida K Yamada T. Randomized comparison of surgical stress and the nutritional status between laparoscopy-assisted and open distal gastrectomy for gastric cancer. Ann Surg Oncol. (2014) 21:1983–90. doi: 10.1245/s10434-014-3509-9 24499830

[130] Fujii K Sonoda K Izumi K Shiraishi N Adachi Y Kitano S. T lymphocyte subsets and Th1/Th2 balance after laparoscopy-assisted distal gastrectomy. Surg Endosc. (2003) 17:1440–4. doi: 10.1007/s00464-002-9149-3 12820059

[131] Shu ZB Cao HP Li YC Sun LB. Influences of laparoscopic-assisted gastrectomy and open gastrectomy on serum interleukin-6 levels in patients with gastric cancer among Asian populations: a systematic review. BMC Gastroenterol. (2015) 15:52. doi: 10.1186/s12876-015-0276-4 25928408 PMC4424540

[132] Lv AQ Huang LC Lao WL Song QL Zhou QF Jiang ZM. Effects of different depth of anesthesia on perioperative inflammatory reaction and hospital outcomes in elderly patients undergoing laparoscopic radical gastrectomy. BMC Anesthesiol. (2022) 22:328. doi: 10.1186/s12871-022-01854-8 36284289 PMC9594928

[133] Liu W Wu L Zhang M Zhao L. Effects of general anesthesia with combined epidural anesthesia on inflammatory response in patients with early-stage gastric cancer undergoing tumor resection. Exp Ther Med. (2019) 17:35–40. doi: 10.3892/etm.2018.6898 30651762 PMC6307522

[134] Wang L Liang S Chen H Xu Y Wang Y. The effects of epidural anaesthesia and analgesia on T lymphocytes differentiation markers and cytokines in patients after gastric cancer resection. BMC Anesthesiol. (2019) 19:102. doi: 10.1186/s12871-019-0778-7 31185917 PMC6560762

[135] Kun L Tang L Wang J Yang H Ren J. Effect of combined general/epidural anesthesia on postoperative NK cell activity and cytokine response in gastric cancer patients undergoing radical resection. Hepatogastroenterology. (2014) 61:1142–7. 26158178

[136] Konstantis G Tsaousi G Kitsikidou E Zacharoulis D Pourzitaki C. The immunomodulatory effect of various anaesthetic practices in patients undergoing gastric or colon cancer surgery: A systematic review and meta-analysis of randomized clinical trials. J Clin Med. (2023) 12:6027. doi: 10.3390/jcm12186027 37762967 PMC10531584

[137] Liu R Qin H Wang M Li K Zhao G. Transversus abdominis plane block with general anesthesia blunts the perioperative stress response in patients undergoing radical gastrectomy. BMC Anesthesiol. (2019) 19:205. doi: 10.1186/s12871-019-0861-0 31699052 PMC6839132

[138] Moon J Chun DH Kong HJ Lee HS Jeon S Park J. The intraoperative administration of dexmedetomidine alleviates postoperative inflammatory response in patients undergoing laparoscopy-assisted gastrectomy: A double-blind randomized controlled trial. Biomedicines. (2023) 11:3253. doi: 10.3390/biomedicines11123253 38137474 PMC10741238

[139] Zhu M Qi Y He H Zhang S Mei Y. Effect of quadratus lumborum block on postoperative cognitive function in elderly patients undergoing laparoscopic radical gastrectomy: a randomized controlled trial. BMC Geriatr. (2021) 21:238. doi: 10.1186/s12877-021-02179-w 33836651 PMC8033654

[140] Lao WL Song QL Jiang ZM Chen WD Zheng XH Chen ZH. The effect of oxycodone on post-operative pain and inflammatory cytokine release in elderly patients undergoing laparoscopic gastrectomy. Front Med (Lausanne). (2021) 8:700025. doi: 10.3389/fmed.2021.700025 34540861 PMC8440846

[141] Kim Y Hwang W Cho ML Her YM Ahn S Lee J. The effects of intraoperative esmolol administration on perioperative inflammatory responses in patients undergoing laparoscopic gastrectomy: a dose-response study. Surg Innov. (2015) 22:177–82. doi: 10.1177/1553350614532534 24803523

[142] Zang YF Li FZ Ji ZP Ding YL. Application value of enhanced recovery after surgery for total laparoscopic uncut Roux-en-Y gastrojejunostomy after distal gastrectomy. World J Gastroenterol. (2018) 24:504–10. doi: 10.3748/wjg.v24.i4.504 PMC5787785 29398871

[143] Tang A Zhou S. Analysis on the application value of goal-directed fluid therapy in patients undergoing laparoscopy-assisted radical gastrectomy with fast-track anesthesia. Am J Transl Res. (2021) 13:5174–82. PMC8205733 34150106

[144] Miyachi T Tsuchiya T Oyama A Tsuchiya T Abe N Sato A. Perioperative oral administration of cystine and theanine enhances recovery after distal gastrectomy: a prospective randomized trial. JPEN J Parenter Enteral Nutr. (2013) 37:384–91. doi: 10.1177/0148607112458798 22972879

[145] Cao W Zheng C Xu X Jin R Huang F Shi M. Clostridium butyricum potentially improves inflammation and immunity through alteration of the microbiota and metabolism of gastric cancer patients after gastrectomy. Front Immunol. (2022) 13:1076245. doi: 10.3389/fimmu.2022.1076245 36466862 PMC9714544

[146] Fu H Li B Liang Z. Effect of enteral immunonutrition compared with enteral nutrition on surgical wound infection, immune and inflammatory factors, serum proteins, and cellular immunity in subjects with gastric cancer undergoing a total gastrectomy: A meta-analysis. Int Wound J. (2022) 19:1625–36. doi: 10.1111/iwj.13763 PMC9615293 35352476

[147] Cheng Y Zhang J Zhang L Wu J Zhan Z. Enteral immunonutrition versus enteral nutrition for gastric cancer patients undergoing a total gastrectomy: a systematic review and meta-analysis. BMC Gastroenterol. (2018) 18:11. doi: 10.1186/s12876-018-0741-y 29338698 PMC5771223

[148] Sedman PC Ramsden CW Brennan TG Giles GR Guillou PJ. Effects of low dose perioperative interferon on the surgically induced suppression of antitumour immune responses. Br J Surg. (1988) 75:976–81. doi: 10.1002/bjs.1800751012 3265347

[149] Romano F Cesana G Berselli M Gaia Piacentini M Caprotti R Bovo G. Biological, histological, and clinical impact of preoperative IL-2 administration in radically operable gastric cancer patients. J Surg Oncol. (2004) 88:240–7. doi: 10.1002/jso.20155 15565596

[150] Rajala P Kaasinen E Raitanen M Liukkonen T Rintala E. Perioperative single dose instillation of epirubicin or interferon-alpha after transurethral resection for the prophylaxis of primary superficial bladder cancer recurrence: a prospective randomized multicenter study–FinnBladder III long-term results. J Urol. (2002) 168:981–5. doi: 10.1016/S0022-5347(05)64556-9 12187204

[151] Schneider C von Aulock S Zedler S Schinkel C Hartung T Faist E. Perioperative recombinant human granulocyte colony-stimulating factor (Filgrastim) treatment prevents immunoinflammatory dysfunction associated with major surgery. Ann Surg. (2004) 239:75–81. doi: 10.1097/01.sla.0000103062.21049.82 14685103 PMC1356195

[152] Wang X Tang S Cui X Yang J Geng C Chen C. Cytokine-induced killer cell/dendritic cell-cytokine-induced killer cell immunotherapy for the postoperative treatment of gastric cancer: A systematic review and meta-analysis. Med (Baltimore). (2018) 97:e12230. doi: 10.1097/MD.0000000000012230 PMC6133452 30200148

[153] Mu Y Zhou CH Chen SF Ding J Zhang YX Yang YP. Effectiveness and safety of chemotherapy combined with cytokine-induced killer cell /dendritic cell-cytokine-induced killer cell therapy for treatment of gastric cancer in China: A systematic review and meta-analysis. Cytotherapy. (2016) 18:1162–77. doi: 10.1016/j.jcyt.2016.05.015 27421742

[154] Faghfuri E Shadbad MA Faghfouri AH Soozangar N. Cellular immunotherapy in gastric cancer: adoptive cell therapy and dendritic cell-based vaccination. Immunotherapy. (2022) 14:475–88. doi: 10.2217/imt-2021-0285 35232264

[155] Entezam M Sanaei MJ Mirzaei Y Mer AH Abdollahpour-Alitappeh M Azadegan-Dehkordi F. Current progress and challenges of immunotherapy in gastric cancer: A focus on CAR-T cells therapeutic approach. Life Sci. (2023) 318:121459. doi: 10.1016/j.lfs.2023.121459 36720453

[156] Long B Qin L Zhang B Li Q Wang L Jiang X. CAR T−cell therapy for gastric cancer: Potential and perspective (Review). Int J Oncol. (2020) 56:889–99. doi: 10.3892/ijo.2020.4982 32319561

[157] Wu X Huang S. HER2-specific chimeric antigen receptor-engineered natural killer cells combined with apatinib for the treatment of gastric cancer. Bull Cancer. (2019) 106:946–58. doi: 10.1016/j.bulcan.2019.03.012 31711572

[158] Yost KE Satpathy AT Wells DK Qi Y Wang C Kageyama R. Clonal replacement of tumor-specific T cells following PD-1 blockade. Nat Med. (2019) 25:1251–9. doi: 10.1038/s41591-019-0522-3 PMC6689255 31359002

[159] Wu TD Madireddi S de Almeida PE Banchereau R Chen YJ Chitre AS. Peripheral T cell expansion predicts tumour infiltration and clinical response. Nature. (2020) 579:274–8. doi: 10.1038/s41586-020-2056-8 32103181

[160] Valpione S Galvani E Tweedy J Mundra PA Banyard A Middlehurst P. Immune-awakening revealed by peripheral T cell dynamics after one cycle of immunotherapy. Nat Cancer. (2020) 1:210–21. doi: 10.1038/s43018-019-0022-x PMC7046489 32110781

[161] Ohmura H Yamaguchi K Hanamura F Ito M Makiyama A Uchino K. OX40 and LAG3 are associated with better prognosis in advanced gastric cancer patients treated with anti-programmed death-1 antibody. Br J Cancer. (2020) 122:1507–17. doi: 10.1038/s41416-020-0810-1 PMC7217874 32203221

