# Supplementary material for: Dysregulation of systemic immunity and its clinical application in gastric cancer
Source: Front Immunol. 2024 Sep 5;15:1450128. doi: 10.3389/fimmu.2024.1450128 (PMC11410619; doi:10.3389/fimmu.2024.1450128)
Supplement: Supplementary file 1 [file Table1.docx]

Supplementary Material

**Supplementary Table S1. Perturbations of systemic immunity induced by gastric cancer.**

| Study | Cases | Healthy | Analyzed indices | Compared with healthy subjects | PMID |
| --- | --- | --- | --- | --- | --- |
| Hou Y, 2023 | 77 | 30 | IL-2, IL-4, IL-6, IL-10, IFN-γ, TNF-α, IL-17A | Higher IL-6, IL-10, TNF-α | 38022654 |
| Qi Q, 2023 | 52 | 31 | IL-1β, IL-6, IL-12, IL-17, IL-8, IL-5, IL-2, IL-4, IL-10, TNF-α, IFN-α, IFN-γ | Higher IL-6, IL-1β, IFN-γ, IL-17, and IL-12p70; Lower IL-4 | 36516540 |
| Guo GH, 2023 | 139 | 40 | GM-CSF, IFN-γ, IL-1β, IL-2, IL-4, IL-6, IL-8, IL-10, MCP-1, TNF-α | Higher TNF-α | 36185564 |
| Gu JH, 2021 | 180 | 170 | IL-35 | Higher IL-35 | 33044052 |
| Ge X, 2021 | 55 | 52 | MIC-1 | Higher MIC-1 | 32918498 |
| Bednarz-Misa I, 2020 | 64 | 39 | IL-1β, IL-1ra, IL-4, IL-5, IL-6, IL-7, IL-8, IL-9, IL-10, IL-12(p70), IL-13, IFN-γ, IP-10, EOX-1, FGF-2,G-CSF, GM-CSF, MCP-1, MIP-1α, MIP-1β, PDGF-BB, RANTES, TNF-α, VEGF-A | Higher IL-1β, IL-4, IL-6, IFN-γ, PDGF-BB, G-CSF; Lower IL-1ra, IL-12(p70), IL-13, MCP-1, RANTES | 32630408 |
| Sun X, 2019 | 142 | 98 | IFN-γ, IL-1β, IL-2, IL-4, IL-6, IL-8, IL-10, IL-12p70, IL-13, TNF-α, GM-CSM, IL-1α, IL-5, IL-7, IL-12/23 p40, IL-15, IL-16, IL-17α, TNF-β, VEGF | Higher IL-12/IL-23P40, IL-15, IL-8, IL-10 | 30847182 |
| Li J, 2018 | 176 | 204 | IL-6, IL-8, TNF-α | Higher IL-6, IL-8, TNF-α | 29881236 |
| Zhong F, 2015 | 47 | 33 | IL‑1β, IL‑17A and IL‑23 | Higher IL‑1β, IL‑17A and IL‑23 | 26352729 |
| Saito H, 2015 | 30 | 12 | IL-17 | Higher IL-17 | 25869448 |
| Epplein M, 2013 | 180 | 358 | IL-1β, IL-2, IL-4, IL-6, IL-8, IL-10, TNF-α, and IFN-γ | Higher IL-8 | 24052422 |
| Ikeguchi M, 2009 | 90 | 9 | IL-6 and IL-10 | Higher IL-6 | 19562463 |
| Onouchi H, 2002 | 76 | 45 | MCP-1 | Lower MCP-1 | 12190098 |
| Sánchez-Zauco N, 2017 | 162 | 201 | IL-1β, IL-6, TNF-α, IL-10, and MCP-1, IL-8, IFN-γ, TGF-β | Higher IL-1β, IL-6, IFN-γ, and IL-10; Lower MCP-1 | 28558708 |
| Szkaradkiewicz A, 2010 | 42 | 20 | IL-10, TGF-β1 | Higher IL-10, TGF-β1 | 20445748 |
| Macrì A, 2006 | 10 | 15 | IL-1β, IL-8, TNF-α | Higher L-1β, IL-8, TNF-α | 16766394 |
| Zhang Y, 2022 | 36 | 18 | IL-21, IL-10, CXCL13 | Higher CXCL13 level were significantly increased | 36339942 |
| Arii K, 2000 | 84 | NA | Neutrophils | Normal phagocytosis, reduced superoxide generation | 10690623 |
| Li JC, 2022 | 63 | 13 | NETs | More likely NET formation | 36051331 |
| Li C, 2022 | 41 | 10 | NETs | Higher NETs | 35664777 |
| Zhu T, 2021 | 51 | 16 | NETs | Higher NETs | 34013374 |
| Yu D, 2024 | 52 | 34 | Neutrophil-derived exosomes (Neu-Exo) | Higher Neu-Exo miR-223-3p | 38199579 |
| Wang Z, 2017 | 62 | 45 | Monocytes | Upregulated TIM-3 | 27911104 |
| Chang LL, 2012 | 5 | 9 | Monocyte-derived DCs | Lower CD40 and IL-10 expression | 22526791 |
| Mao FY, 2018 | 43 | 45 | MDSCs | Higher CD45+CD33lowCD11bdim MDSCs | 29988030 |
| Xu DP, 2016 | 124 | 130 | DC-10 | Higher HLA-G+ DC-10 | 26773190 |
| Liu W, 2018 | 32 | 35 | pDCs, mDC1s | Higher pDCs, mDC1s | 29552142 |
| Chen J, 2014 | 8 | 8 | DCs, NK cells | Lower DCs, NK cells; Decreases production of TNF-α, IL-2 and T-bet | 25550889 |
| Yu H, 2023 | 30 | 30 | DCs, T cells | Higher pDCs, CCR9-pDCs, Tregs, ICOS^+^ Tregs, ICOS^-^ Tregs | 37126939 |
| Huang XM, 2014 | 51 | 30 | pDCs, Tregs | Higher pDCs, Tregs, and ICOS(+) Tregs | 24261990 |
| Jung YJ, 2023 | 42 | 13 | B10 cells; Plasmablasts; Plasma cells | Higher B10 cells; Lower Plasmablasts | 37946227 |
| Hu HT, 2019 | 22 | 22 | IL-10-producing B cells | No significant differences | 31574287 |
| Lundin BS, 2007 | 8 | 7 | T cells | Higher amounts of IL-10 production when stimulated with H. pylori antigens | 17826353 |
| Xu J, 2021 | 32 | 23 | CD8^+^ T cells | Lower TLR7 expression and percentage of TLR2^+^ cells | 34620075 |
| Zhong C, 2019 | 26 | 26 | CD8^+^ T cells | Higher IFN-γ^+^IL10^+^CD8^+^ T cells; Lower IFN-γ^+^IL10^-^CD8^+^ T cells, IFN-γ^+^CD8^+^ T cells | 31260655 |
| Zhong F, 2015 | 47 | 33 | Th17, Tc17 and γδT17 cells | Higher Th17, γδT17 cells; Lower Tc17 | 26352729 |
| Saito H, 2015 | 30 | 12 | Tc17 cells | Higher Tc17 cells | 25869448 |
| Zhuang Y, 2012 | 103 | 57 | Tc17 cell | Higher Tc17 cells | 22710190 |
| Li H, 2023 | 29 | 29 | Lymphocyte PD-1, Tregs | Higher lymphocyte PD-1 and FoxP3^+^ Tregs | 37970473 |
| Zhang Y, 2022 | 36 | 18 | Tfh cells, Breg cells | Higher Tfh cells, Breg cells | 36339942 |
| Wang WW, 2015 | 107 | 45 | Breg cells | Higher Breg cells | 26378021 |
| Li J, 2024 | 20 | 20 | CD3^+^ T cells, CD4^+^ T cells, CD8^+^ T cells, CD19^+^ B cells, and lymphocytes | Lower CD3^+^ T cells, CD4^+^ T cells, CD8^+^ T cells, CD19^+^ B cells, and lymphocytes | 38259671 |
| Wang K | 50 | 30 | B cells, Treg cells, MDSCs, activated CD4^+^ T cells, naive CD4^+^ T cells, memory CD4^+^ T cells, activated CD8^+^ T cells, monocytes | Higher IL-35-producing B cells and positively associated with stage | 29742730 |
| Chen J, 2021 | 90 | 45 | N#, L#, M#, N%, L%, M%, Hb, Hct, RDW, PLT#, PDW, MPV, MPV/PLT, MLR, NLR, PLR | Higher M#, M%, RDW-CV, MPV, PDW, MLR, NLR, N#, N%, PLR; Lower Hb, L%, Hct | 34429765 |
| Han B, 2018 | 30 | 30 | NK cells | Lower NK cells expressing NKp30, NKp46, NKG2D, and DNAM-1; | 30255106 |
| Lee J, 2017 | 261 | 48 | NK cells | Lower IFN-γ production | 29050291 |
| Wang Z, 2015 | 62 | 32 | NK cells | Higher TIM-3 expression | 26214042 |
| Peng YP, 2013 | 31 | 31 | NK cells | Lower NKG2D, NKp30, NKp46, and perforin positive NK cells; higher KIR3DL1 positive NK cells | 24138752 |
| Lindgren Å, 2011 | 13 | 15 | NK cells | Impaired IFN-γ production | 21255568 |
| Szkaradkiewicz A, 2010 | 42 | 20 | NK cells | Decreased cytotoxic function | 20445748 |
| Hou Y, 2023 | 77 | 30 | TLC, NLR, PLR, LMR | Higher NLR, PLR; Lower TLC, LMR | 38022654 |
| Zhang J, 2023 | 125 | 125 | PLR, NLR, and SII | Higher PLR, NLR, and SII | 37064093 |
| Fang T, 2020 | 2606 | 3219 | NLR, PLR | Higher NLR and PLR | 32211444 |
| Uzunoglu H, 2023 | 152 | 152 | SII, NLR | Higher SII, NLR | 36910431 |
| Zhao Q, 2022 | 773 | 2368 | PDW, NLR, PLR | Higher PDW, NLR, PLR | 36451446 |
| Wu Y,, 2018 | 161 | 157 | NLR, PLR | Higher NLR, PLR | 29476736 |
| Zhang L, 2021 | 155 | 162 | MLR | Higher MLR | 34162569 |
| Lian L, 2015 | 162 | 30 | PLR, NLR | Higher PLR, NLR | 26444485 |

**Supplementary Table S2. Table S2. Changes of systemic immunity during traditional therapy for gastric cancer.**

| Study | Cases | Treatment | Analyzed indices | Time-point | Findings | PMID |
| --- | --- | --- | --- | --- | --- | --- |
| Servis D, 2008 | NA | Surgery | CRP, IL-2, IL-6, IL-10, TNF-α, IFN-γ, cortisol, WBC | Baseline; 3h, 1d, 2d after surgery | Increase: WBC, IL-4, IL-6, IL-10 and cortisol peaked at 3h. IL-6 concentration was higher after longer operations | 19102411 |
| Sun HL, 2014 | 60 | Surgery | IL-2, IL-6, IL-10, sIL-2R | 1.5h, 1d, 2d, 3d after surgery | 1.5h; only IL-6 increase; 1d: IL-2 decrease, IL-6, IL-10, sIL-2R increase; 2d, starting to return but still high; 3d, only IL-6 to baseline | 25295719 |
| Shito M, 1998 | 20 | Surgery | IL-1, TNF, IL-6 | 1d, 3d, 5d, 7d after surgery | Increase: IL-6 | 9537718 |
| Jung YJ | 36 | Surgery | B10 cells, plasmablasts, plasma cells | 1-3 m after surgery | Increase: plasmablasts, plasma cells; Decrease: B10 cells | 37946227 |
| Gryglewski A, 2007 | Mice | Surgery | Mesenteric lymph node γδT cells | After surgery | Surgical stress causes accumulation of γδT cells to suppress the cell-mediated response via TGF-beta | 17612560 |
| Okuno K, 1999 | 49 | Surgery | T cells, NK cells | After surgery | T-cell mediated functions were suppressed. Decreased T-cell subsets (CD 3^+^, 4^+^, 8^+^) and increased NK cell subsets (CD 16^+^, 57^+^) | 11957066 |
| Hou Y, 2023 | 41 | Immunochemotherapy | IL-2, IL-4, IL-6, IL-10, IFN-γ, TNF-α, IL-17A | After 2 cycles | Increase: IL-2, IL-4, IL-10, and IFN-γ | 38022654 |
| Hou Y, 2023 | 36 | Chemotherapy | IL-2, IL-4, IL-6, IL-10, IFN-γ, TNF-α, IL-17A | After 2 cycles | Decrease: IL-2, TNF-α, IFN-γ, and IL-17A | 38022654 |
| Ohmura H, 2020 | 30 | Immunotherapy | IL-2, IFN-γ, TNF-, IL-4, IL-5, IL-6, IL-10, IL-13, IL-17A, IL-17F, IL-21, IL-22, IL-9, T cells, B cells, NK cells, DCs, and monocytes | Baseline, after 1 cycle | Significant increases in activated central/effector memory, activated effector T cells, and activated T-helper 1 subsets | 32203221 |
